# Biosignature stability in space enables their use for life detection on Mars

**DOI:** 10.1126/sciadv.abn7412

**Published:** 2022-09-07

**Authors:** Mickael Baqué, Theresa Backhaus, Joachim Meeßen, Franziska Hanke, Ute Böttger, Nisha Ramkissoon, Karen Olsson-Francis, Michael Baumgärtner, Daniela Billi, Alessia Cassaro, Rosa de la Torre Noetzel, René Demets, Howell Edwards, Pascale Ehrenfreund, Andreas Elsaesser, Bernard Foing, Frédéric Foucher, Björn Huwe, Jasmin Joshi, Natalia Kozyrovska, Peter Lasch, Natuschka Lee, Stefan Leuko, Silvano Onofri, Sieglinde Ott, Claudia Pacelli, Elke Rabbow, Lynn Rothschild, Dirk Schulze-Makuch, Laura Selbmann, Paloma Serrano, Ulrich Szewzyk, Cyprien Verseux, Dirk Wagner, Frances Westall, Laura Zucconi, Jean-Pierre P. de Vera

**Affiliations:** ^1^ German Aerospace Center (DLR), Institute of Planetary Research, Planetary Laboratories Department, Rutherfordstr. 2, 12489 Berlin, Germany.; ^2^ Heinrich-Heine-Universität (HHU), Institut für Botanik, Universitätsstr. 1, 40225 Düsseldorf, Germany.; ^3^ German Aerospace Center (DLR), Institute of Optical Sensor Systems, Rutherfordstr. 2, 12489 Berlin, Germany.; ^4^ AstrobiologyOU, Faculty of Science, Technology, Engineering and Mathematics, The Open University, Milton Keynes, MK7 6AA, UK.; ^5^ Microbial Geoecology and Astrobiology, Department of Ecology and Environmental Sciences, Umeå university, Linnaeus väg 6, 901 87 Umeå, Sweden.; ^6^ Department of Biology, University of Rome Tor Vergata, Via della Ricerca Scientifica, 00133, Rome, Italy.; ^7^ Department of Ecological and Biological Sciences (DEB), University of Tuscia, Largo dell’Università snc, 01100 Viterbo, Italy.; ^8^ Departamento de Observación de la Tierra, Instituto Nacional de Técnica Aeroespacial (INTA), Torrejón de Ardoz-28850, Madrid, Spain.; ^9^ European Space Agency (ESA), European Space Research and Technology Centre (ESTEC),, Noordwijk, Netherlands.; ^10^ University of Bradford, University Analytical Centre, Division of Chemical and Forensic Sciences, Raman Spectroscopy Group, West Yorkshire, UK.; ^11^ Leiden Observatory, Laboratory Astrophysics, Leiden University, Leiden, Netherlands.; ^12^ George Washington University, Space Policy Institute, Washington, DC 20052, USA.; ^13^ Freie Universitaet Berlin, Experimental Biophysics and Space Sciences, Institute of Experimental Physics; Arnimallee 14, 14195 Berlin, Germany.; ^14^ Faculty of Earth and Life Sciences, Vrije Universiteit Amsterdam, De Boelelaan 1081-1087, 1081 HV, Amsterdam, Netherlands.; ^15^ CNRS Centre de Biophysique Moléculaire, UPR-4301, Rue Charles Sadron, CS80054, 45071 Orléans Cedex 2, France.; ^16^ Biodiversity Research/Systematic Botany, University of Potsdam, Maulbeerallee 1, D-14469 Potsdam, Germany.; ^17^ Department Technology Assessment and Substance Cycles, Leibniz- Institute for Agriculture Engineering and Bioeconomy, Max-Eyth-Allee 100, D-14469 Potsdam, Germany.; ^18^ Institute for Landscape and Open Space, Eastern Switzerland University of Applied Sciences, Seestrasse 10, 8640 Rapperswil, Switzerland.; ^19^ Institute of Molecular Biology and Genetics of NASU, Acad. Zabolotnoho str.150, 03680, Kyiv Ukraine.; ^20^ Centre for Biological Threats and Special Pathogens (ZBS 6), Robert Koch Institute, Nordufer 20, 13353 Berlin, Germany.; ^21^ German Aerospace Center (DLR), Institute of Aerospace Medicine, Radiation Biology Department, Linder Höhe, 51147 Köln, Germany.; ^22^ Research and Science Department, Italian Space Agency (ASI), Via del Politecnico snc, 00133, Rome, Italy.; ^23^ NASA Ames Research Center, Mail Stop 239-20, P.O. Box 1, Moffett Field, CA 94035-0001, USA.; ^24^ Department of Molecular Biology, Cell Biology and Biochemistry, Brown University, 185 Meeting Street, Providence, RI 02912, USA.; ^25^ Technical University Berlin, ZAA, Hardenbergstr. 36, D-10623 Berlin, Germany.; ^26^ Section Geomicrobiology, German Research Centre for Geosciences (GFZ), Telegrafenberg, 14473 Potsdam, Germany.; ^27^ Department of Experimental Limnology, Leibniz-Institute of Freshwater Ecology and Inland Fisheries (IGB), 12587, Stechlin, Germany.; ^28^ Mycological Section, Italian Antarctic National Museum (MNA), 16121 Genoa, Italy.; ^29^ Helmholtz Centre for Polar and Marine Research, Alfred Wegener Institute (AWI), Telegrafenberg, 14473 Potsdam, Germany.; ^30^ Institute of Environmental Technology, Environmental Microbiology, Technical University Berlin, Ernst-Reuter-Platz 1, Berlin, 10587 Berlin, Germany.; ^31^ Center of Applied Space Technology and Microgravity (ZARM), University of Bremen, Am Fallturm 2, 28359, Bremen, Germany.; ^32^ Institute of Geosciences, University of Potsdam, Karl-Liebknecht-Str. 24, 14476, Potsdam, Germany.; ^33^ German Aerospace Center (DLR), Microgravity User Support Center (MUSC), Linder Höhe, 51147 Köln, Germany.

## Abstract

Two rover missions to Mars aim to detect biomolecules as a sign of extinct or extant life with, among other instruments, Raman spectrometers. However, there are many unknowns about the stability of Raman-detectable biomolecules in the martian environment, clouding the interpretation of the results. To quantify Raman-detectable biomolecule stability, we exposed seven biomolecules for 469 days to a simulated martian environment outside the International Space Station. Ultraviolet radiation (UVR) strongly changed the Raman spectra signals, but only minor change was observed when samples were shielded from UVR. These findings provide support for Mars mission operations searching for biosignatures in the subsurface. This experiment demonstrates the detectability of biomolecules by Raman spectroscopy in Mars regolith analogs after space exposure and lays the groundwork for a consolidated space-proven database of spectroscopy biosignatures in targeted environments.

## Teaser

Biomolecules were preserved under martian conditions simulated on the ISS and their analysis supports the search for life on Mars.

## INTRODUCTION

NASA’s Curiosity rover recently confirmed the presence of organic molecules on Mars (*1*, *2*): from the simplest (methane) to more complex carbon compounds (e.g., thiophenic, aromatic, and aliphatic) in drill samples from Mars’ Gale Crater. These findings raise hopes of discovering biosignatures in the form of more complex, organic molecules synthesized biologically, by the current and upcoming Mars rovers: Perseverance, part of NASA’s Mars 2020 mission (successfully landed on 18th of February 2021) and the Rosalind Franklin rover and its Pasteur instruments suite, part of ESA and Roscosmos’s ExoMars mission (scheduled for launch in September 2022). Both rovers will be able to perform Raman spectroscopic measurements (*3*–*5*), a rapid and nondestructive technique, particularly suited for the identification of organic biosignatures within their mineralogical context on Earth (*6*, *7*). However, the martian environment is expected to affect the stability and detectability of biosignatures because of both the physicochemical conditions at the surface and the composition of the martian regolith. Ultraviolet radiation (UVR), ionizing radiation, and oxidizing compounds may destroy detectable traces from the upper centimeters to meters of the martian surface (*8*). Although the regolith that surrounds potential biomolecules may offer some protection and stabilization (*9*), it also tends to increase the Raman signal background level, decreasing the signal-to-noise ratio, and thus the chance of biomolecule detection (*10*, *11*).

While amino acids and DNA (*12*, *13*) were previously exposed to low Earth orbit (LEO) to assess their detectability and stability, they were not analyzed by Raman spectroscopy. Thus, to test the potential of Raman spectroscopy to identify biomolecules on Mars, we exposed seven biomolecules (β-carotene, chlorophyllin, naringenin, quercetin, melanin, cellulose, and chitin; Table 1) mixed with two martian regolith analogs [the Phyllosilicatic Mars Regolith Simulant (P-MRS) and the Sulfatic Mars Regolith Simulant (S-MRS); table S1] to simulated Mars conditions in LEO. These biomolecules were selected because of their relevance as putative martian biosignatures and for their detectability using Raman spectroscopy. They represent ubiquitous biogenic compounds found in a large number of extremophilic organisms with paramount structural (cellulose and chitin) or protective (quercetin, naringenin, β-carotene, and melanin) capacities, which might also be compatible with martian life, if it exists. A full rationale for the choice of biomolecules is discussed in Materials and Methods with additional information in Supplementary Text.

**Table 1.** Biogenic compounds characterized by Raman spectroscopy.

**Biogenic compound**	**Substance class**	**Biological significance**	**Chemical formula**	**Mol. weight (g/mol)**	**Supplier**	**Purity**
**β-Carotene**	Carotenoid	Antioxidant in bacteria, plants, and fungi/accessory photo-pigment/ quenches excess photo-energy	C_40_H_55_	536.89	Fluka	≥97%
**Chlorophyllin (Na-Cu-salt)**	pPorphyrin derivative	Synthetic derivative/derivate for chlorophyll as geoporphyrin	C_34_H_31_N_4_O_6_Na_3_Cu	724.19	Sigma-Aldrich	com. grade
**Naringenin**	Flavonoid	Common antioxidant in plants/scavenges free radicals	C_15_H_12_O_5_	272.26	Alfa Aesar	97%
**Quercetin (dihydrate)**	Flavonoid	Common antioxidant in plants/scavenges free radicals	C_15_H_10_O_7_	338.27	Sigma-Aldrich	≥98%
**Melanin**	Tyrosine derivative	Most groups of organisms, esp. fungi and animals/ UV-protective pigment/effective energy dissipation	Variable	Variable	Sigma	≥97%
**Cellulose**	Polysaccharide	Cell wall component of plants, algae, oomycetes/ component of biofilms/polymer of d-glucose	(C_6_H_10_O_5_)_n_	variable	Sigma-Aldrich	≥97%
**Chitin**	Polysaccharide	Component of fungal cell walls and of arthropod exoskeletons/ polymer of *N*-acetylglucosamine	(C_8_H_13_O_5_N)_n_	~400,000	Roth	Com. grade

Several critical factors of the martian environment that are expected to have a large impact on biosignatures—notably the photonic and ionic radiation—cannot be simulated on Earth with high fidelity (*14*). For this, we used ESA’s EXPOSE-R2 platform (*15*), which exposed four experiments outside the International Space Station (ISS) for 469 days. The BIOlogy and Mars EXperiment (BIOMEX) (*16*, *17*) hosted extremophiles and their cellular components, mixed with analogs of lunar and martian regolith, and exposed them to space and simulated Mars-like conditions (LEO’s solar and galactic cosmic radiation, extreme temperature cycles, space vacuum, a Mars-like atmosphere, with and without UVR). The mission was launched on 24 July 2014 with the Progress 56P and lasted for 696 days from launch to landing. After samples were returned to Earth with the Soyuz 45S on 18 June 2016, the stability of the biomolecules and the survival of intact organisms were assessed. Postflight Raman analyses were conducted under oxic and anoxic conditions, with two different Raman setups (at 532 and 514 nm), and qualitatively and quantitively compared to preflight and nonirradiated ground controls.

## RESULTS AND DISCUSSION

### Characterization of biomolecules and regolith analog mixtures

**Fig. 1.**
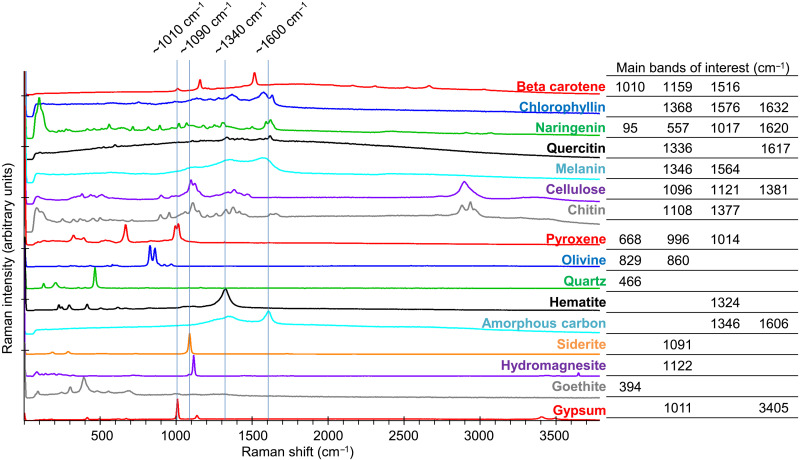
Raman spectra of investigated biogenic compounds and martian regolith analogs. Stacked Raman spectra recorded at 532-nm excitation wavelength with overlapping regions, indicated by blue lines, and main bands of interest (details in fig. S1).

During the preparatory phase of the experiment, biomolecules and regolith analogs were characterized separately by Raman spectroscopy. The position and intensity of the Raman bands agreed with the available literature (Fig. 1, fig. S1B, and Table 2). β-Carotene, as a member of the carotenoid family, presents a conjugated polyene skeletal backbone. Its spectrum showed mainly one medium and two strong Raman bands: at ~1010 cm^−1^ for the in-plane rocking modes of the CH_3_ groups attached to the polyene chain, at ~1159 cm^−1^ for the C─C stretching and C─H deformation, and at ~1516 cm^−1^ for the C═C stretching (*18*, *19*). The copper sodium salt of chlorophyllin is a derivative of chlorophyll a, with the cyclopentanone ring of chlorophyll being oxidized and the phytyl ester group hydrolyzed. Its Raman spectrum resembled that of metallochlorin and metalloporphyrin, with the main bands at ~1368 and ~1577 cm^−1^ being assigned to the totally symmetric ring stretching modes (*20*). Naringenin is a flavonoid belonging to the flavanones subclass with three hydroxy groups located at the 4′, 5, and 7 carbon atoms. It showed a very rich and complex Raman spectrum due to numerous potential vibrations. One of the strongest features was found around ~1622 cm^−1^ corresponding to C─C aromatic ring stretching (*21*). Quercetin is also a flavonoid with a similar structure to naringenin. Its main vibrations are therefore similar, with prominent bands in the ~1617 cm^−1^ region, corresponding to C═C stretching and O─H bending, and in the ~1336 cm^−1^ region, also attributed to O─H bending (*22*). Melanin is a more complex heteropolymer with varying units, and its spectrum was very close to disordered graphite, with the well-known D and G bands at ~1380 and ~1580 cm^−1^, respectively. The first band was tentatively attributed to the stretching of the C─C bonds within the rings of the aromatic melanin monomers, while the second might be due to the linear stretching of the aromatic rings themselves (*23*). Cellulose is a linear polymer of β-d-glucose; however, its Raman spectrum did not correspond to that of its monomer and presents broader bands, with features at ~1098 cm and ~1384 cm^−1^ corresponding to C─O and C─C stretches and CH_2_ and CH_2_OH deformations, respectively (*18*). Chitin is a polymer of *N*-acetyl-d-glucosamine, and, as for cellulose, its Raman spectrum presented broader features than that of its monomer but with very similar regions to other saccharides (~1108 cm^−1^ for C─O and C─C stretches and ~1377 cm^−1^ for CH_2_ and CH_2_OH deformations) (*18*).

**Table 2.** Main Raman bands of investigated biomolecules compared with literature. w, weak; m, medium; s, strong; mw, medium weak; vs, very strong; sh, shoulder. Main bands used for identification are highlighted in bold.

**Biogenic compound**	**Measured Raman bands and approximate intensities (cm^−1^ with 532-nm excitation)**	**Raman bands and approximate intensities from literature**		
		**Raman bands (cm^−1^)**	**Laser (nm)**	**Reference**
**β-Carotene**	**1010 m**; **1159 s**; 1193 w; 1214 vw; 1273 vw; **1516 vs**; 2163 vw; 2311 w; 2518 vw; 2666 w; 3024 vw	**1008 m**; **1156 vs**; 1190 m; 1211 m; 1270 w; 1280 w/sh; 1353 w; 1394 w; 1448 w; **1515 vs**	785	(*18*)
**Chlorophyllin (Na-Cu-salt)**	127 w; 754 w; 829 w; 952 w; 991 w; 1137 m; 1158 m; 1225 m; 1280 m; **1368 s**; **1576 vs**; 1632 s	781 m; 905 w; 925 w; 1003 w; 1139 w; 1229 w; 1281 w; **1365 s**; 1383 m; 1467 w; 1508 w; **1595 s**; 1635 s	1064	(*44*)
**Naringenin**	**95 vs**; 228 w; 275 w; 414 w; 456 w; 507 w; **557 m**; 714 w; 816 w; 891 m; 970 w; **1017 m**; 1065 m; 1175 m; 1218 w; 1251 m; 1314 m; 1498 w; 1588 m; **1620 m**; 2905 vw; 3068 vw	273 m; 526 w; **610 s**; **1000 m**; 1191 w; 1247 m; 1509 m; 1586 m; **1613 m**	1064	(*21*)
**Quercetin**	105 w; 489 w; 521 w; 598 m; 845 w; 1108 w; 1160 w; **1336 s**; 1369 m; 1402 m; 1438 m; 1471 m; 1549 m; 1585 m; **1617 vs**	604 w; 640 w; 661 w; 686 w; 721 w; 785 w; 843 w; 942 w; 1013 w; 1175 w; 1216 w; 1268 m; 1315 m; **1328 s**; 1357 m; 1371 m; 1398 m; 1410 w; 1440 m; 1463 w; 1531 w; 1548 m; 1596 m; 1604 w; **1609 s**; 1662 m	1064	(*22*)
**Melanin**	**1348 vs; 1564 vs**	**1387 s; 1592 s**	514.5	(*23*)
		**1370 s; 1590 s**	514	(*51*)
**Cellulose**	86 w; 379 m; 435 w; 508 w; 898 w; 971 w; **1096 vs**; **1121 s**; 1340 m; **1381 m**; 1467 w; 2892 s	380 m; 436 w; 458 m; 493 m; 520 mw; 577 mw; 896 m; 969 w; 998 w; 1046 mw; 1061 m; **1096 vs**; **1120 s**; 1147 m; 1266 mw; 1337 m; **1379 m**; 1413 m; 1461 m	785	(*18*)
**Chitin**	81 vs; 252 w; 327 w; 369 w; 397 w; 452 w; 499 w; 566 w; 648 vw; 711 vw; 897 m; 953 m; 1061 m; **1108 s**; 1147 m; 1206 w; 1265 w; 1327 m; **1377 m**; 1414 m; 1621 w; 1657 w; 2707 vw; 2883 m; 2937 s; 2961 m	366 m; 396 m; 460 m; 498 m; 711 mw; 895 m; 955 m; 1059 m; **1107 s**; 1149 m; 1205 mw; 1262 m; 1328 m; **1371 m**; 1414 m; 1449 mw; 1626 m; 1656 m	785	(*18*)

The position and intensity of the biomolecules and regolith analogs’ Raman bands illustrate the abovementioned challenges for the unambiguous identification of biosignatures in regolith: Some of the most intense (and therefore detectable) bands of biomolecules and minerals fall within, or close to, the same spectral range (Fig. 1) (*11*). Furthermore, space instruments have much lower spatial resolution than do laboratory micro-Raman. The diameter of the laser spot is 50 μm for the Raman laser spectrometer (RLS) on ExoMars’ Rosalind Franklin rover (*3*). Mars2020’s Perseverance rover carries two Raman systems: the Scanning Habitable Environments with Raman and Luminescence for Organics and Chemicals (SHERLOC) with a 50-μm laser spot size (*4*) and the remote-Raman on SuperCam with a laser spot size from ~1 mm at a 1.4-m distance to ~4 mm at a 7-m distance (*24*). In contrast, the laser spot diameter was 2.5 μm of the micro-Raman used in the present study. Depending on the grain size distribution, each Raman spectrum acquired on Mars could thus be composed of a mixture of a larger number of components (organic and mineral), with lower signal-to-noise ratios, potentially inhibiting the identification of individual components. Even for bigger grains, the quality of the derived spectra will depend on the number of mineral phases in the analyzed spot.

### Exposure conditions

**Fig. 2.**
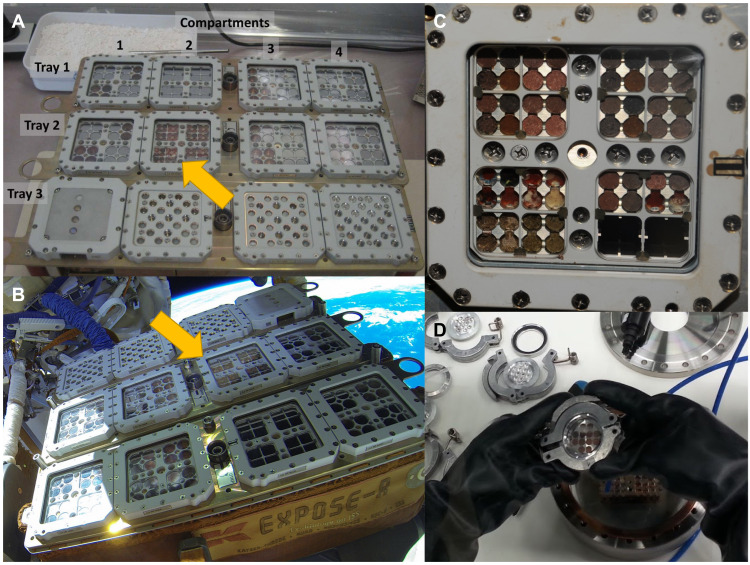
Sample setup on the EXPOSE-R2 platform. (**A**) Sample arrangement of the biogenic compounds in tray 2 compartment 2 in the EXPOSE-R2 hardware at MUSC/DLR, Cologne (arrow) (credits: MUSC/DLR/Elke Rabbow). (**B**) EXPOSE-R2 hardware outside the Zvezda module of the ISS with tray 2, compartment 2 in the center (arrow). Photograph taken at EVA (extravehicular activity) #40 after removal of the EXPOSE-R2 protective cover by cosmonauts A. Samokutyaev and M. Surayev (credits: ESA/Roscosmos). (**C**) Close-up of tray 2, compartment 2 of EXPOSE-R2 after exposure inside the ISS, with the P-MRS and S-MRS samples in the left and right upper half, respectively (credits: ESA/Roscosmos). (**D**) Transfer of samples in air-tight containers under anoxic atmosphere for Raman measurements.

The biomolecules in the Mars regolith analogs were exposed to LEO conditions using the EXPOSE-R2 facility. Compartment 2 of tray 2 of the facility contained three layers of sample carriers (Fig. 2). The Top layer was exposed to solar radiation (with filters cutting off wavelengths below 200 nm, comparable to the martian atmosphere) for 469 days, while the layers below (middle and bottom) were shielded from solar radiation and served as dark controls (*16*). The total UVR dose received by the upper layer ranged from 316 to 587 MJ m^−2^, which corresponds to approximately 200 to 400 days under the average UVR flux at the surface of the martian equator, at the vernal equinox (*25*). All samples remained in a Mars-like atmosphere for 722 days (starting from before launch) and were exposed during flight to extreme temperature cycles (ranging from −20.5° to +47.2°C) and LEO’s ionizing radiation (*15*) that differs from that of Mars due to the influence of Earth’s magnetic field in LEO (*14*, *26*).

### Qualitative analysis of biomolecule detection after exposure

**Fig. 3.**
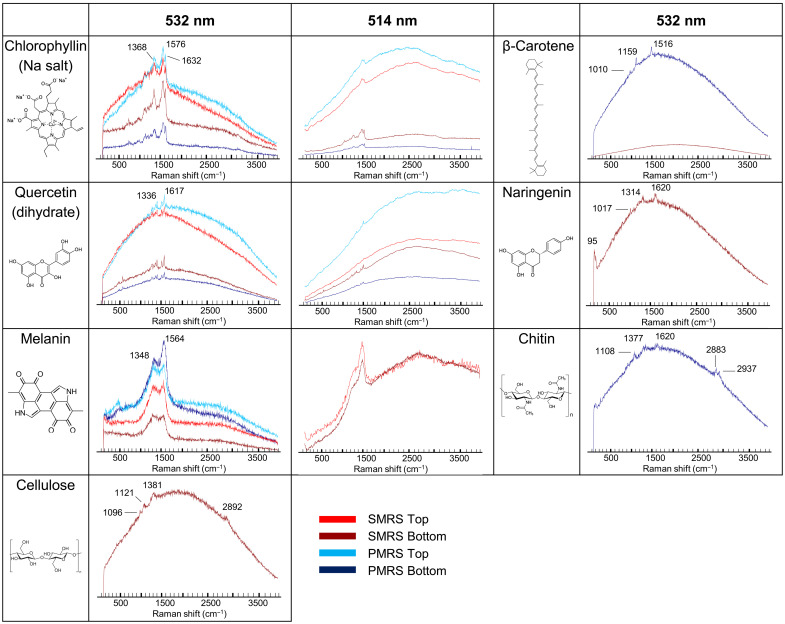
Detectability of biomolecular spectra, qualitative analysis. Maximum intensity raw spectra obtained for biomolecules mixed with S-MRS and P-MRS exposed in the upper sample tray (S-MRS Top, red; P-MRS Top, light blue) and in the lower sample tray (S-MRS Bottom, dark red; P-MRS Bottom, dark blue). Spectra were acquired with a 532-nm excitation wavelength (DLR Berlin) and with a 514-nm excitation wavelength (OU Milton Keynes). β-Carotene, naringenin, cellulose, and chitin were detected only on a few spots and only in the samples for which a spectrum is shown. Pure S-MRS and P-MRS samples did not yield any biomolecule spectrum. The same scale is applied for the spectral intensity.

Of the seven tested biomolecules, only three could be detected in the UVR irradiated samples for both mineral substrates (Fig. 3): chlorophyllin, quercetin, and melanin. When samples were shielded from UV, all seven biomolecules were detectable, although bands for β-carotene, naringenin, cellulose, and chitin were visible only in a small fraction of all acquired spectra. These results differ to some extent from those obtained, in the framework of the BIOMEX experiment, with biomolecules within intact organisms. Carotenoids, for instance, were shown to resist space exposure and in ground-based simulations when contained in organisms, even when the host organisms were no longer viable. Carotenoids spectra were recorded on the UV-exposed bacterium *Deinococcus radiodurans*, exposed in LEO in the BIOMEX project, but with low signal-to-noise ratios and in low instances (*27*), on UV-shielded samples of the cyanobacterium *Chroococcidiopsis* sp. exposed in LEO on EXPOSE-R (*28*), and even on the cyanobacterium *Nostoc* sp. after very high dose gamma irradiation (*29*). These differences can be explained by the different mixture of carotenoid molecules present in these organisms (deinoxanthin in *D. radiodurans*; β-carotene, zeaxanthin, echinenone, and myxixanthophyll among others in *Nostoc* spp.) compared to the single selected β-carotene in this study and by the shielding and potential stabilization provided by cellular components. Bacterial cellulose was also exposed as a structural component of pellicle produced by acetic acid bacteria from a kombucha microbial community (Komagataeibacter, former Gluconacetobacter), and, although not analyzed by Raman spectroscopy, Fourier-transform infrared spectroscopy showed that its structural integrity was unchanged (*30*). No biomolecular spectrum was recorded in the negative controls (biomolecule-free, P-MRS, or S-MRS samples) showing the absence of cross-contamination within the exposure platform.

### Quantitative analysis via filtering and clustering methods

The preservation of the different biomolecules was quantified as signal coverage, defined as the fraction of all recorded spectra for which the biomolecule signals could be identified. Two separate methods were used to differentiate biomolecular spectra from mineral spectra and background noise: manual identification through filtering using the WITec Project FIVE software (fig. S2) and chemometric methods relying on Hierarchical Cluster Analysis (HCA) using the Unscrambler X software. For the HCA, we focused on the dataset acquired using the 532-nm excitation wavelength, as it is most relevant as a reference for Raman spectrometers on Mars that will operate at this wavelength. Furthermore, differences in data acquisition and preprocessing would have led to systematic discrepancies between the 532- and 514-nm datasets. For filtering, both datasets were used. Filtering and HCA gave similar results: The overall difference was 5% (table S2). As cellulose, β-carotene and chitin signals were detected only in UV-shielded samples and with very low coverage (below 1%), these molecules were excluded from the quantitative analyses.

Exposure to ambient air during analysis was not found to negatively affect the detectability of the biomolecules. Given the large number of conditions and the unknown population-level SD for each of them, we refrained from considering individual pairs; taken as a whole, the results obtained from the oxic and Mars-like anoxic sets (see Materials and Methods) did not differ significantly from each other (table S3). The results were consequently pooled to provide a better coverage of the samples.

**Fig. 4.**
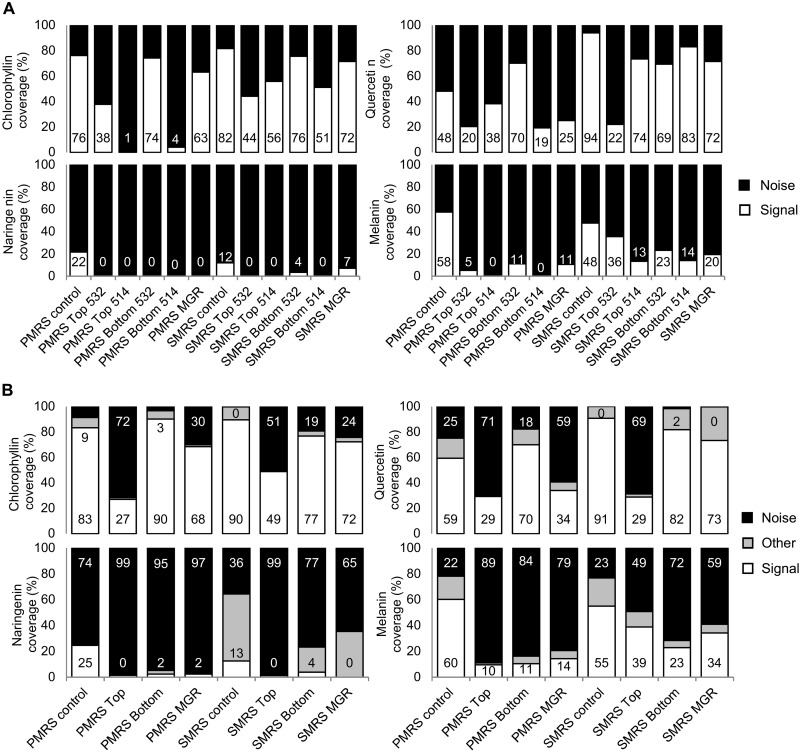
Coverage of signal, quantitative analysis. (**A**) Results of filter/manual counting for the four biomolecules with the most signal (chlorophyllin, naringenin, quercetin, and melanin) analyzed with a 532-nm excitation wavelength (DLR Berlin) and with a 514-nm excitation wavelength (OU Milton Keynes) for the Top (UV exposed), Bottom (UV shielded), MGR, and control samples. Positive signal is shown in white, and actual coverage percentage is indicated at the bottom of the bar. (**B**) Results of HCA for the four biomolecules with the most signal (chlorophyllin, naringenin, quercetin, and melanin) divided into three classes: signal (white, with percentage indicated at the bottom of the bar), other (gray), and noise (black, with percentage indicated at the top of the bar). Labels of the bar charts are indicated below the second rows.

Data from preflight controls, as well as samples kept under anoxic conditions for the whole duration of the mission [Mission Ground Reference (MGR)], were included in this analysis. The filtering analysis results show that the extent to which detectability was affected by UVR depends on both the substrates (P-MRS or S-MRS) and as mentioned above, the Raman setup (Fig. 4A). Discrepancies between Raman setups are, besides the different excitation wavelengths (532 versus 514 nm), due to differences in instrument resolution, noise, and wavelength-dependent luminescence background emission in some of the samples (see Fig. 3). The difference was particularly large for samples mixed with the P-MRS analog, most likely due to the high luminescence background of the clay minerals that masked the signal of interest.

The 532-nm recorded spectra were further classified by HCA into three clusters: signal of interest, other spectral features, and noise (Fig. 4, A and B, and figs. S4 to S7). Chlorophyllin and quercetin signals were most detectable overall and were recorded under all test conditions, although with differences in signal coverage (Fig. 4, A and B). For chlorophyllin, UVR exposure reduced the signal coverage from 77 to 49% in S-MRS and from 90 to 27% in P-MRS. Similarly, for quercetin, UV exposure decreased signal coverage by 50% (S-MRS) and 40% (P-MRS). Naringenin was detected in UV-shielded samples (Bottom) only, with a coverage of 2 to 4%. The case of melanin is slightly more complex. Although its spectrum is close to that of amorphous carbon, the HCA method successfully separated both signals on S-MRS (fig. S7). However, on the P-MRS analog, the width of the bands comprising its Raman signature, combined with a low signal-to-noise ratio, hindered its detection: The identification procedure that was followed for the quantitative analysis failed to assign a large fraction of the spectra showing melanin bands to the “signal” class (fig. S7). Optimization of the measurement parameters could help to address these limitations. In the present study, we could nonetheless document a drop in signal coverage between controls and flight samples: by 50% (regardless of UV exposure) on P-MRS and by 20 to 30% on S-MRS. Melanin was also detected in organisms, namely, the Antarctic fungus *Cryomyces antarcticus*, exposed in LEO as part of BIOMEX (*31*).

### The composition of the martian regolith analogs affected the detection of the biosignatures

Particularly challenging was the proximity or overlap between biogenic and mineral bands: Some of the latter covered signals of interest entirely. For instance, the broad and intense band of hematite at ~1320 cm^−1^ (abundant in S-MRS) falls within the same range as prominent bands of chlorophyllin, quercetin, melanin, cellulose, and chitin (Fig. 1). The amorphous carbon signature at ~1350 and ~1600 cm^−1^ present in P-MRS also covered some of the principal bands of biogenic compounds (Fig. 1 and figs. S4 to S7). Furthermore, the luminescence background of both minerals (but mainly of P-MRS) was exacerbated by UV in the Top layer samples (Fig. 3, light blue and light red curves). The overall signal coverage was higher in S-MRS, regardless of UV exposure (Fig. 4, A and B), which can be ascribed in large part to the high intrinsic fluorescence of P-MRS’s clay components (*10*). Although clays are often considered the preferred matrix for finding preserved organic molecules on Mars (*1*, *32*), these results emphasize the challenge posed by minerals to Raman spectroscopy–based biomolecule detection on the martian surface. Therefore, it would also be worth exploring the detection of biomolecules in other mineral matrices, such as silica (*33*), which have also been identified at the landing sites for both the Perseverance and Rosalind Franklin rovers (*34*, *35*). Alternatively, improvements to standard Raman spectroscopy, such as time-resolved Raman spectroscopy, show promising results to analyze highly fluorescent samples (*36*) but are still in need of miniaturization for space missions. Last, the use of internal and external standards coupled with data normalization models has also recently been shown to be able to provide quantitative information on organic mineral mixtures and is an interesting approach for current and future missions (*37*).

### UV exposure largely reduced the detectability by Raman spectroscopy of biosignatures

That was the case even for the most resistant biomolecules, chlorophyllin and quercetin, whose coverage dropped by 30 to 50%. The lack of measurements at intermediate time points—a common constraint for LEO experiments (*14*)—limits the conclusions that can be drawn about the dynamics of biosignature degradation. Our results nonetheless demonstrate that signals would become undetectable after at most a few years under the average UV flux of the martian equator at the vernal equinox (assuming a degradation linear with the cumulated UV dose, within 700 to 1200 days; assuming first-order kinetics, within 1200 to 4500 days). On the contrary, the signal preservation in UV-shielded (Bottom layer) samples was remarkable with signal coverage close to the controls for chlorophyllin and quercetin, despite the temperature cycles, Mars-like atmosphere, and ionizing radiation. This is a central result of our study and corroborates the relevance—in the search for traces of life—of the martian subsurface, which the Rosalind Franklin rover will reach with a 2-m drill (*38*).

### Summary and outlook

Our results are the first reported on the Raman signatures of isolated, LEO-exposed biomolecules. They corroborate the idea that Raman spectroscopy, a fast and nondestructive technique, is highly relevant to the search for traces of life on Mars—especially in the UV-shielded subsurface. The biomolecules tested in BIOMEX represent key resistance roles in terrestrial organisms and are therefore suitable analogs of complex molecules in putative martian life playing similar roles. A broader perspective could be achieved by testing a wider range of biomolecules; ideally, with the implementation of a systematic Raman (bio)signature database. This database would be a valuable consolidated and space-proven reference for future search-for-life missions on Mars and elsewhere in our solar system.

## MATERIALS AND METHODS

### Selection of the biogenic compounds

The present selection of biogenic compounds, their substance classes, biological roles, chemical formulas, molecular weight, supplier, and purity are given in Table 1. Among the 26 BIOMEX samples (*16*), seven of them contained biomolecules selected for their detectability using Raman spectroscopy and their relevance as putative martian biosignatures: β-carotene, chlorophyllin, naringenin, quercetin, melanin, cellulose, and chitin. Some of these play a key role in photoautotrophic metabolism (carotenes and chlorophylls) and structural integrity (cellulose and chitin) of cells, but our chief criterion for selecting these molecules was the advantage they can confer upon organisms when it comes to resisting the harsh martian environment; β-carotene, melanin, quercetin, and naringenin, for instance, are potent scavengers of stress-induced radicals. In addition, these molecules were found in a wide range of organisms hosted in the BIOMEX experiment (*16*): A significant number of bacteria contain carotenoids and some bacteria and cyanobacteria produce cellulose (*39*); algae, cyanobacteria, lichens, and bryophytes contain carotenoids (*11*), chlorophyll, and flavonoids (such as quercetin and naringenin) (*40*, *41*); and fungi and lichens contain melanin (*42*) and chitin (*43*). Chlorophyll was replaced with its derivative, the copper sodium salt of chlorophyllin, which produces very distinguishable Raman spectra when excited at 532 nm (*44*). Well-preserved fossil chlorophyll derivatives are often found in sedimentary organic matter (*45*). While the exact same molecules are not to be expected in martian life, they are representative of key resistance roles in terrestrial organisms and are, therefore, suitable analogs of complex molecules in putative martian life playing similar roles. Considering the similarities found between early Earth and early Mars environments in terms of water availability and UVRs (*46*), these functions and properties might also be compatible with putative martian organisms, if life ever appeared on Mars (*47*–*49*). See Supplementary Text for a full rationale on the biogenic compounds’ selection.

### Sample selection and preparation

For more realistic measurements, biomolecules were embedded in one of two martian regolith analogs (table S1): The P-MRS and the S-MRS. P-MRS and S-MRS are analogous to, respectively, the clay-rich regolith from the Noachian and the sulfate-rich regolith from the Hesperian and Amazonian (*10*, *16*, *17*). All the biogenic compounds were commercial grade compounds of ≥97% purity (see Table 1). The two martian analog mineral mixtures used were developed in the framework of the BIOMEX project by the Museum für Naturkunde Berlin (see table S1) according to the data of Mars missions [see (*10*)] to simulate early acidic Mars and late basic Mars surface lithosphere composition (table S1). The P-MRS simulated igneous rocks altered by hydrous fluids (pH neutral) to clays of the smectite group, such as montmorillonite and chamosite and the clay mineral kaolinite. Siderite and hydromagnesite were used for carbonates and for the basaltic subsurface rocks. The S-MRS mimicked a more acidic environment with sulfate deposits: It included igneous rocks, anhydrous iron oxides, goethite, and gypsum. Minerals were mechanically crushed, and only fragments smaller than 1 mm were used for the mineral mixtures.

The selected biogenic compounds were mixed with S-MRS and P-MRS powders of 25- to 1000-μm grain size at 5% (w/w) to enable complete interaction between the compounds, potentially degrading martian substrates, and—during exposure or its simulation—the applied environmental conditions. The mixture was pressed into ⌀ 6 mm pellets of 0.4 g each at 6 t for 15 min in a PP-10 pellet press (Retsch, Germany).

### Exposure conditions outside the ISS

Samples were exposed to Mars simulated conditions for close to 16 months (469 days) outside the ISS, on two layers as “Top” (exposed to sun light) and “Bottom” (dark control in space) in the tray 2 of the EXPOSE-R2 platform (Fig. 2, A to C). Tray 2 (Mars tray) was filled with a Mars-like gas mixture composed of 95.55% CO_2_, 2.70% N_2_, 1.60% Ar, 0.15% O_2_, ~370 parts per million H_2_O, at a pressure of 980 Pa. It was covered by a quartz window (transmission: λ > 170 nm) supplemented with a cutoff filter (transmission: λ > 200 nm) to approach the solar wavelength spectrum at the surface of Mars. UV, ionizing radiation, and temperature sensors recorded the exposure conditions in real time or upon return (*15*).

#### Solar irradiation

The total solar dose (UV + photosynthetically active radiation: 200 to 700 nm) received by the compartment 2 of tray 2 ranged between 8340 and 3890 MJ m^−2^ according to the samples’ positions.

#### Temperature

The highest recorded temperature was +47.2°C, the lowest was −20.5°C, and the average was +18.5°C. The strong temperature variations were caused by the constantly changing attitude of the orbiting EXPOSE facility toward the sun.

#### Cosmic radiation

The total mission dose of the BIOMEX samples was 200 to 230 mGy. With a mission duration of 696 days, this translated into a daily dose of 290 to 330 μGy.

#### Mission ground reference

An identical set of flight samples was exposed on the ground in the Planetary and Space Simulation Facilities (PSI) chamber at German Aerospace Center (DLR) Cologne for the whole duration of the mission with an identical Mars-like gas mixture at 980 Pa.

### Mission timeline

BIOMEX spent approximately 23 months in space (696 days from launch to landing), including close to 16 months (469 days) exposed to UVR. An outgassing period of 62 days (for the samples under space conditions in Tray 1), where samples were outside the ISS but protected against solar irradiation by a sun shield, preceded UV exposure (*15*).

12-06-2014 Sample integration completed at Microgravity User Support Center (MUSC)/DLR, Cologne

23-07-2014 Launch

18-08-2014 Trays placed outside the ISS

22-10-2014 Sun shield removed; start of UV exposure

03-02-2016 Trays moved back into the ISS

18-06-2016 Landing

24-06-2016 Sample deintegration at MUSC/DLR, Cologne

### Raman measurements

To increase the robustness of our results, two different Raman setups were used, operated by separate teams. The excitation wavelengths (532 and 514 nm) are the same or close to the wavelength that will be used by instruments on both the Perseverance and Rosalind Franklin rovers (532 nm for SuperCam on Perseverance, and for RLS on Rosalind Franklin, Perseverance also carries SHERLOC, a deep UV Raman instrument with a 248.6-nm wavelength).

Raman spectra were obtained with a confocal WITec alpha 300 system, at the DLR Berlin, consisting of a microscope equipped with a 10× long working distance objective with a 0.25 numerical aperture, a piezo-driven scan table, a UHTS 300 spectrometer with an ultrafast EMCCD detector, and a frequency-doubled Nd:YAG laser. The excitation wavelength of the laser was 532 nm, the spot diameter at the sample was ~2.5 μm, and the spectral resolution of the spectrometer was 4 to 5 cm^−1^ with a 600-lines/mm grating. Integration time and laser power were varied according to the investigated biomolecule (for β-carotene, quercetin, and chitin, the laser power was 1 mW and the integration time was 1 s; for chlorophyllin, the laser power was 0.7 mW and the integration time was 2 s; for naringenin and melanin, the laser power was 1.25 mW and the integration time was 1 s; and for cellulose, the laser power was 7 mW and the integration time was 2 s) to produce spectra with a sufficient signal-to-noise ratio, to prevent sample damage/degradation and detector saturation according to extensive preflight analyses and more recent work (*10*, *50*). A minimum of five 200-μm line scans with 100 spectra each were acquired per sample at an equal distance from the pellet margin (fig. S1A). Additional measurements were performed as a time series and as single spectra.

The second Raman system was a Horiba Jobin-Yvon LabRam HR800 spectrometer, at the Open University (OU) Milton Keynes, equipped with a 514-nm laser, a 600-lines/mm grating, and a 10× objective with a 0.25 numerical aperture, which gives a spot diameter of 2.5 μm. The laser power used ranged between 0.6 and 0.73 mW at the sample, and LabSpec 6 software was used to collect the data.

### Anoxic and oxic measurements

To avoid the exposure of the samples to Earth’s atmosphere upon their return and thus potential oxidizing reactions, spectra were first acquired under anoxic conditions. Upon their deintegration at MUSC/DLR in Cologne, Germany, the samples were kept in an anaerobic bench environment and then transferred to a special carrier maintaining the anoxic conditions for transport to the DLR in Berlin, Germany. They were then transferred to special containers covered by a laser transparent window to allow Raman analyses under the maintained anoxic conditions (Fig. 2D). The long working distance objective of our Raman setup allowed the focusing and collection of backscattered photons through the window at 12-mm distance.

### Preprocessing of Raman data

The spectra were first treated with the WITec Project FIVE for preprocessing to remove the contributions of the cosmic rays and assessed visually and with the help of band filters to count the occurrence of relevant signals. They were then exported in the Unscrambler X (10.3) software for further treatment and data analysis. Several preprocessing techniques were tested to remove luminescence and background contributions as well as unwanted variations related to noise, intensity differences, and other spectral artifacts and to enhance the relevant spectral features. For each molecule, the wavenumber region of interest containing the most dominant bands was selected to reduce the data and processing times. Spectra were lastly baseline-corrected, smoothed using a Gaussian filter transform, and the band areas were normalized. The applied transformations are illustrated in fig. S3 with a test set of 600 spectra (of 6000) for the chlorophyllin samples. First, the raw data are plotted and visually analyzed; then for each molecule, the wavenumber region of interest with the most dominant bands was selected to reduce the data and processing times. For most of the biomolecules tested, this wavenumber region falls between 1000 and 1700 cm^−1^ where all the main bands are present (see fig. S1B) except for naringenin, which has a very dominant band at low wavenumbers (80 to 90 cm^−1^). Several smoothing algorithms were tested to reduce the noise while preserving the spectral features of interest, and a Gaussian filter function was found to produce the best results. To correct for intensity differences and remove background and fluorescence contributions, the spectra were lastly baseline-corrected and area-normalized. Background subtraction functions proved inefficient to treat the very different recorded spectra consistently.

### Clustering analysis

Cluster analysis is an exploratory data analysis tool and an unsupervised methodology that provides a valuable and rapid visualization tool in data mining. HCA is based on using linkage methods to generate clusters in general in the form of a tree diagram (dendrogram) and is fine for the assignment of the results of the clustering as a category variable named class for each spectrum. Several linkage methods and distance measurements were tested to provide the best separation of the spectra with the signals of interest from spectra showing other signals (minerals) or noise. HCA was performed with the Unscrambler X (10.3) software to separate the 500 spectra per sample into classes showing signals of interest, other spectral features pertaining to minerals and noise. HCA complete linkage with absolute correlation showed the best results in separating biomolecular spectral features from unwanted signals and was applied to all the samples (figs. S4 to S7).

### Statistical analyses between anoxic and oxic conditions

Statistical analyses were performed in GraphPad Prism 5.0. No statistical difference was accounted for between the anoxic and oxic sets analyzed by either the filtering or the clustering methods: The paired *t* test *P* values are equal to 0.5429 for the filtering set and 0.3131 for the clustering one. However, a few notable differences can be seen for quercetin P-MRS Top, P-MRS Bottom, and S-MRS Top and for Melanin S-MRS Top and Bottom samples (see table S3). The difference in signal coverage between anoxic and oxic conditions was nevertheless not consistent for all the molecules: While, overall, more positive signals were detected under anoxic conditions for chlorophyllin, it was the reverse for quercetin and naringenin. Melanin samples show a greater variability most probably due to a relatively unreliable signal detection but interestingly with much higher signal coverage in the S-MRS samples under anoxic conditions.

## Supplementary Materials

### This PDF file includes:

Supplementary text Figs. S1 to S7 Tables S1 to S3 References
