# Supplementary material for: Biosignature stability in space enables their use for life detection on Mars
Source: Sci Adv. 2022 Sep 7;8(36):eabn7412. doi: 10.1126/sciadv.abn7412 (PMC9451166; doi:10.1126/sciadv.abn7412)
Supplement: Supplementary file 1 — Supplementary text Figs. S1 to S7 Tables S1 to S3 References [file sciadv.abn7412_sm.pdf]

Science Advances
AAAS

Supplementary Materials for

Biosignature stability in space enables their use for life detection on Mars

Mickael Baqué *et al.*

Corresponding author: Mickael Baqué, mickael.baque@dlr.de; Jean-Pierre P. de Vera, jean-pierre.devera@dlr.de

Sci. Adv. **8**, eabn7412 (2022)
DOI: 10.1126/sciadv.abn7412

This PDF file includes:

Supplementary text
Figs. S1 to S7
Tables S1 to S3
References

Supplementary Text

Rationale for the selection of the biogenic compounds

The selection of the tested compounds focused on their definite biological origin. While numerous nucleic acid compounds (pyrimidines and purines), α- and other amino acids, carboxylic acids, aldehydes, alcohols, and simple sugars as well as (often polycyclic aromatic) hydrocarbons were reported to be formed abiotically in interstellar, circumstellar and protoplanetary clouds(*52*), in meteorites(*53*), as well as on early Earth(*54*) and Mars(*55*), none of the presently selected substances have been reported to date in space or on planetary bodies, thus predestining them as potential biomarkers. Moreover, amino acids and nucleobases have already been characterized by Raman spectroscopic techniques(*56*) and extensively considered in the contexts of astrochemistry/-biology(*57*) and Mars(*58*). Therefore, neither of these substance classes was included in the present study.

Photoprotective pigments, such as carotenoids, have been extensively used as model biosignatures due to their known stability and excellent identification by Raman spectroscopy (especially with a 532 nm laser excitation due to resonance effects): from *in situ* measurements in martian analogue environments to laboratory investigations, even when embedded in the mineral matrix(*59*). Carotenoids and fossil carotenoids (such as carotane) have high preservation potential as shown from terrestrial examples where they were successfully identified with gas chromatography / mass spectrometry (GC/MS) and Raman spectroscopy in 1.44 million-year-old halite brine inclusions(*60*) and in 1.64-billion-year-old samples, respectively(*61*). Carotenoids can thus serve as a signature for both extant life and extinct life, in their diagenetically altered form coined biomarkers(*59*, *62*). In addition, they represent a very large class of pigments, with more than 750 different chemical structures determined to date. Due to their distribution in very diverse organisms, including extremophiles, and the several key functions they serve at the cellular level, it has been proposed that carotenoids played an important role in the early evolution of life on Earth(*63*). Indeed, they have excellent antioxidant properties (*64*), which may have been essential for the highly UV irradiated early Earth organisms(*65*), and may have played an early role in membrane stabilization, prior to fatty acids(*66*).

Similarly, the polymer melanin, present in all domains of terrestrial life, is a common light- and UV-protective constituent of astrobiological models such as *Cryomyces antarcticus*(*67*), *Cryptomyces minterii*, *Rhizocarpon geographicum* and *Buellia frigida*(*68*). These organisms have shown extensive resistance to simulated and real space stressors, in particular ionizing radiation, thanks, in part, to their melanin pigmentation. Interestingly, the insoluble organic matter fraction found in carbonaceous chondrites presents similarities to the primary precursors of bacterial and fungal melanins (allomelanins) and may point towards a very early origin of melanin pigments used by terrestrial, and potentially martian, life(*69*).

The blue-light and UV-protective secondary lichen compound parietin, found in the astrobiologically relevant lichens *Xanthoria elegans*(*70*) and *Fulgensia bracteata*(*43*), and the free radical scavenging flavonoids quercetin and naringenin, are all widely distributed in terrestrial life and play essential roles in UV and ionizing radiation resistance.

Chlorophylls are ubiquitous in light harvesting reactions in terrestrial life. At their central structure is an aromatic ring system with a sequestered metal atom, called a porphyrin. Porphyrins were originally regarded as ideal biomarkers(*71*) due to their high preservation potential in the

geological record, as geoporphyrins(*45*), and the fact that no abiotic formation route had been discovered. New laboratory experiments have however disputed these conclusions and shown their potential abiotic formation under specific conditions, suggesting caution when interpreting potential porphyrin signals(*72*). These results also suggest that precursors of chlorophylls and hemes could have been available to the first protocells, which is consistent with the proposed first functions of porphyrins and related pigments in early terrestrial life as UV-protective rather than light-harvesting molecules(*73*).

The two other selected biogenic compounds are structural polysaccharides. Cellulose, as the most common biopolymer on Earth(*74*), is an extracellular polymeric substance produced by phylogenetically diverse bacteria of the genera *Gluconacetobacter*, *Agrobacterium*, *Pseudomonas*, *Rhizobium, Azotobacter*, *Salmonella*, *Alcaligenes* and *Sarcina*, the sulphur-oxidizing bacteria(*75*), and cyanobacteria(*76*). Cellulose-producing microorganisms inhabit seawater, hot springs, dry-lands and other extreme econiches. Microbial cellulose has remarkable physical properties, explaining its stability under high temperatures and pressures, irradiation, and other stressors providing extensive protection to cellulose-synthesizer organisms(*77*). Cellulose might thus have played an important role in the survival of microbial organisms in the harsh conditions of primordial Earth around 3.5 billion years ago and is presumably one of the oldest native macromolecule found on Earth(*78*). Chitin, one of the main constituents of the cell walls of the astrobiological model fungi and lichens mentioned above, has also been reported in organically preserved fossils(*79*) and might play an important role in biomineralization processes, relevant for biosignatures preservation(*80*).

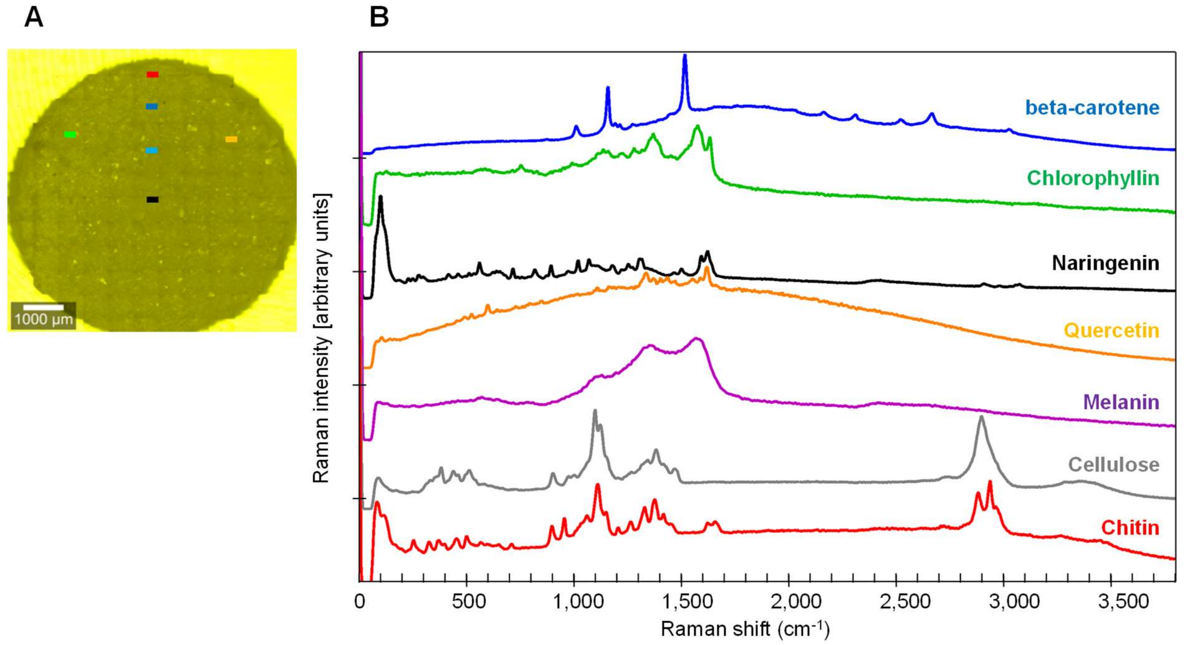

Fig. S1. Raman spectra of pure investigated biogenic compounds: (A), Example of six 200µm-Line scans on a pellet sample. **(B)**, Stacked spectra with different scales acquired with a confocal WITec alpha 300 system at 532 nm excitation wavelength and measurement as reported in Material and Methods.

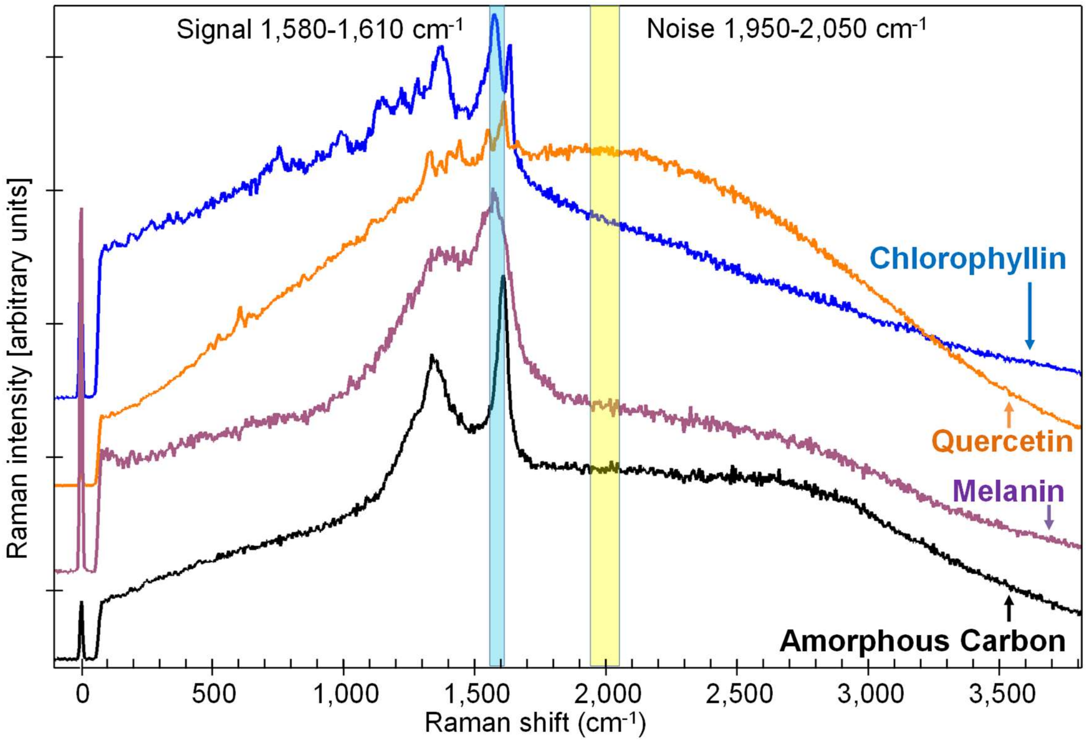

Fig. S2. Filter regions used in the WITec Project FIVE software to identify the biomolecules signal of interest: main region used for signal recognition in blue and region used for noise calculation in yellow. Examples of unprocessed spectra of (from top to bottom): chlorophyllin (in blue), quercetin (in orange), melanin (in purple), and amorphous Carbon (in black).

1	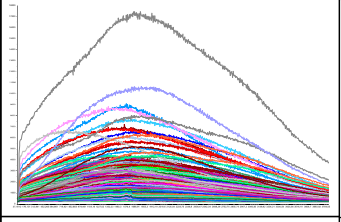	Raw data 0-3,800cm^{-1}
2	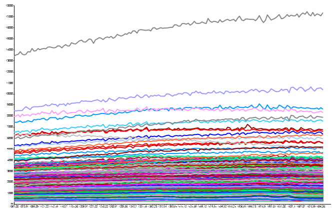	Raw data specifics 1,050-1,700cm^{-1}
3	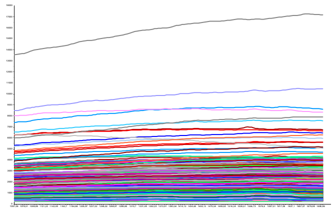	Raw data specifics 1,050-1,700cm^{-1}<br>Smoothed Gaussian Filter segment size 9
4	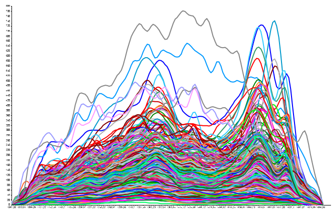	Raw data specifics 1,050-1,700cm^{-1}<br>Smoothed Gaussian Filter segment size 9<br>Baseline Offset & Linear Baseline Correction
5	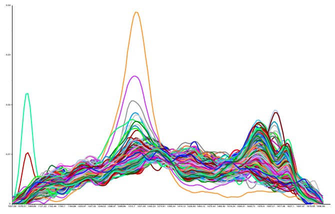	Raw data specifics 1,050-1,700cm^{-1}<br>Smoothed Gaussian Filter segment size 9<br>Baseline Offset & Linear Baseline Correction<br>Area Normalization

Fig. S3. Preprocessing steps illustration on Unscrambler, example on a test set (600 out of 6,000 spectra) for chlorophyllin samples.

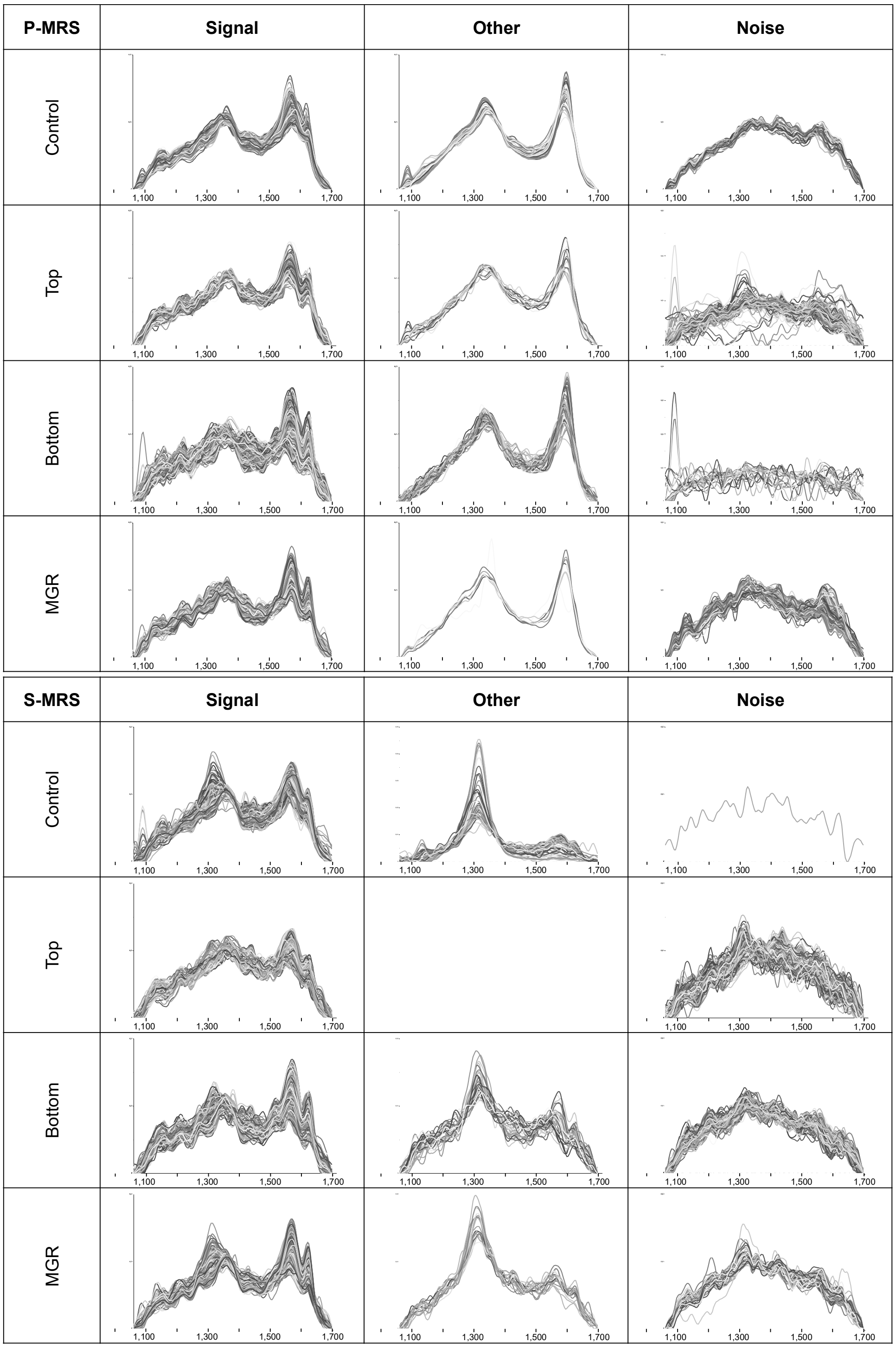

Fig. S4. Identified classes for chlorophyllin on P-MRS and S-MRS. Rows show the different sample types (Control, Top, Bottom, and MGR) while columns show the identified classes (Signal, Other, and Noise). Preprocessed spectra with the region of interest 1,050-1,700 cm^{-1}.

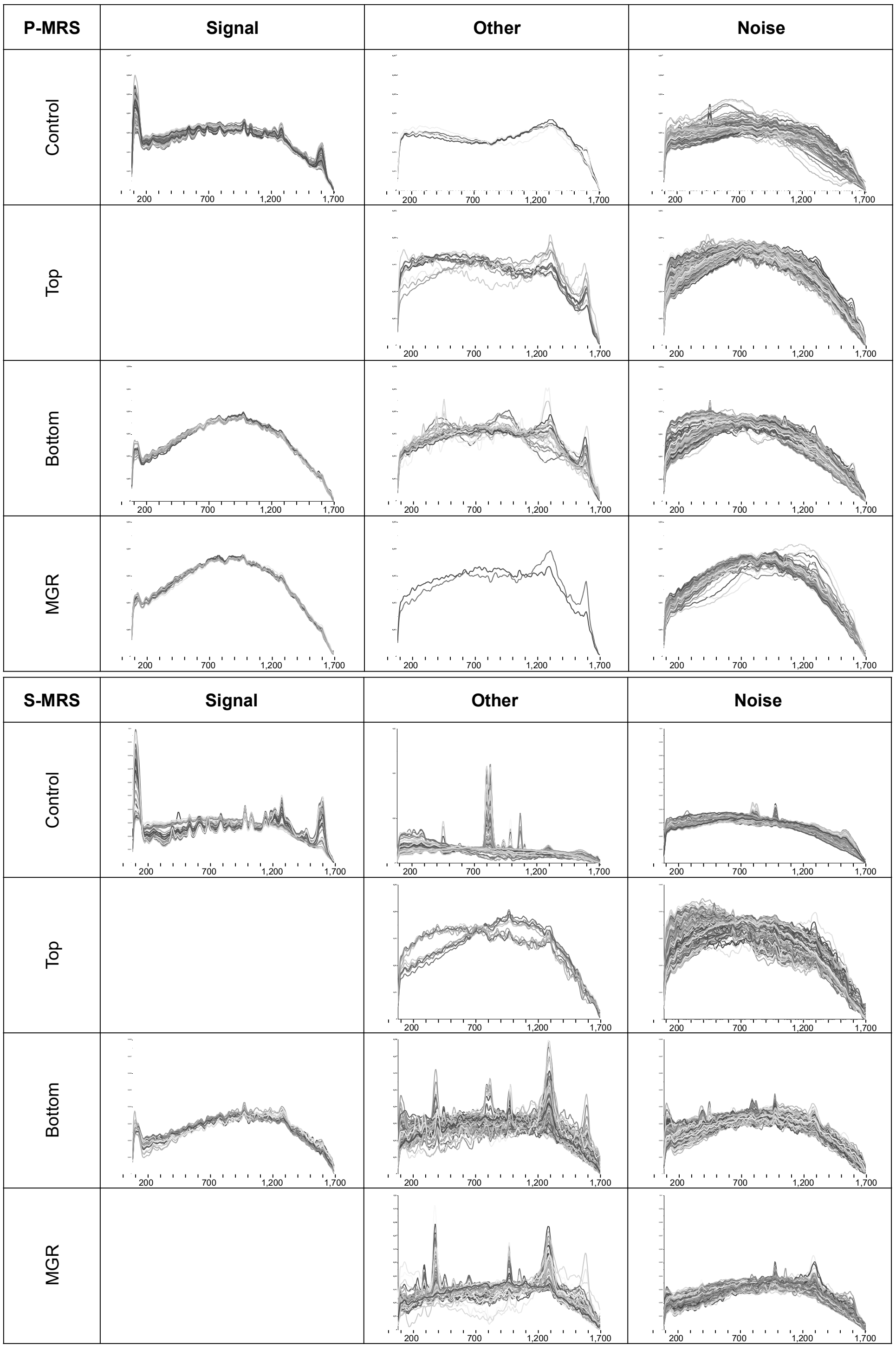

Fig. S5. Identified classes for naringenin on P-MRS and S-MRS. Rows show the different sample types (Control, Top, Bottom, and MGR) while columns show the identified classes (Signal, Other, and Noise). Preprocessed spectra with the region of interest 60-1,700 cm^{-1}.

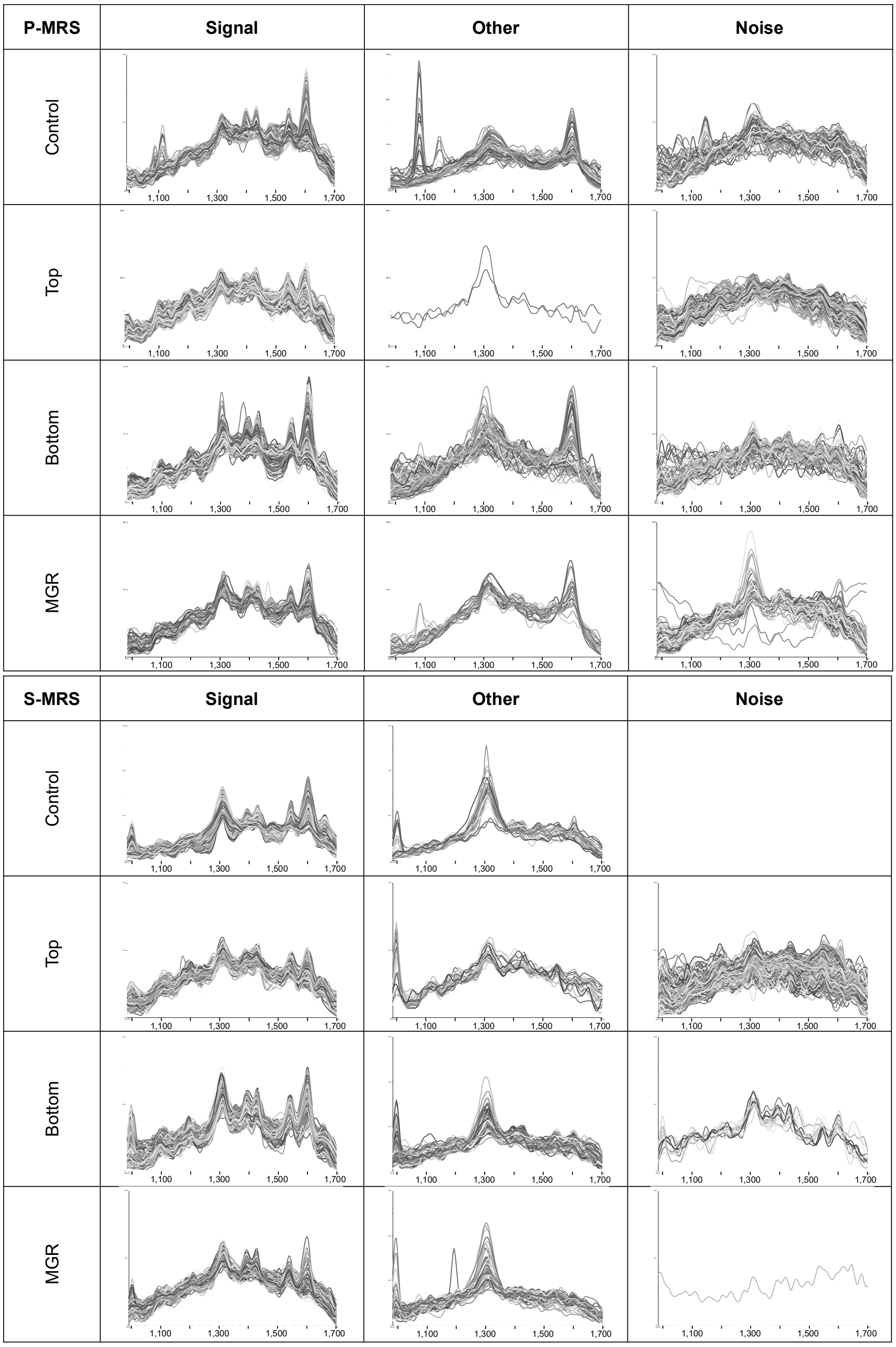

Fig. S6. Identified classes for quercetin on P-MRS and S-MRS. Rows show the different sample types (Control, Top, Bottom, and MGR) while columns show the identified classes (Signal, Other, and Noise). Preprocessed spectra with the region of interest 1,000-1,700 cm^{-1}.

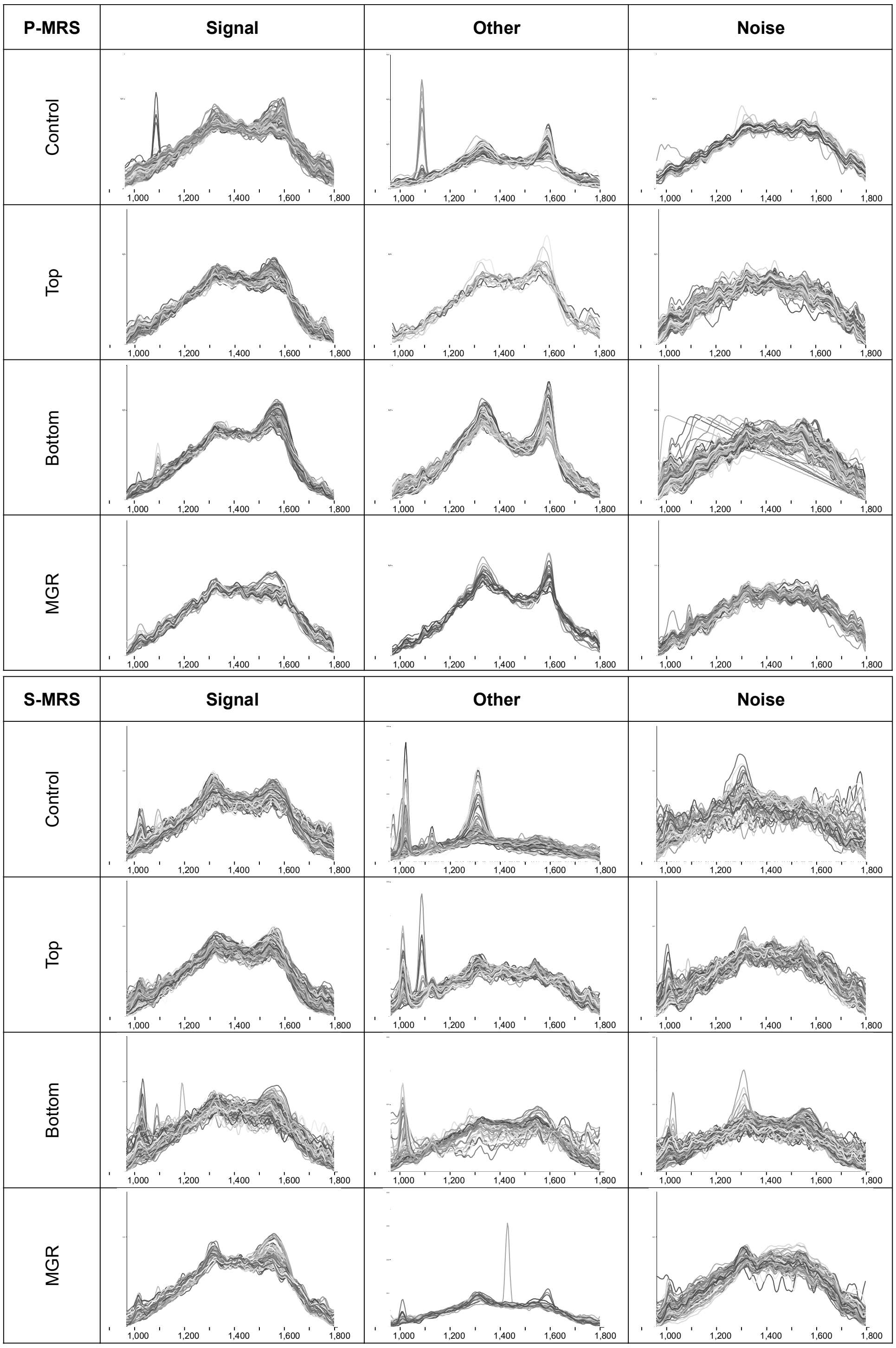

Fig. S7. Identified classes for melanin on P-MRS and S-MRS. Rows show the different sample types (Control, Top, Bottom, and MGR) while columns show the identified classes (Signal, Other, and Noise). Preprocessed spectra with the region of interest 950-1,800 cm^{-1}.

Table S1. Mars regolith simulants (MRS)

Component	Phyllosilicatic MRS (wt/v %) Early Mars	Sulfatic MRS (wt/v %) Late Mars
**Pyroxene, Plagioclase, Amphibole, Ilmenite (Gabbro)**	3	32
**Olivine** $(Mg,Fe)_2SiO_4$	2	15
**Quartz** SiO_2	10	3
**Hematite** Fe_2O_3	5	13
**Montmorrilonite** $[(Na,Ca)_{0.33}(Al,Mg)_2Si_4O_{10}(OH)_2 \times H_2O]$	45	-
**Chamosite** $[(Fe^{2+},Mg,Fe^{3+})5Al(Si_3Al)O_{10}(OH,O)_8]$	20	-
**Kaolinite** $Al_2Si_2O_5(OH)_4$	5	-
**Siderite** $Fe(CO_3)$	5	-
**Hydromagnesite** $Mg_5(CO_3)_4(OH)_2 \times 4H_2O$	5	-
**Goethite** FeO(OH)	-	7
**Gypsum** $Ca(SO_4) \times 2H_2O$	-	30

Table S2. Differences in signal coverage between clustering and filtering methods: differences between combined datasets for clustering and filtering methods.

		Clustering	Filtering	Absolute difference
Chlorophyllin	PMRS Control	83.4%	76.2%	7.2%
	PMRS Top	26.9%	37.7%	10.8%
	PMRS Bottom	90.2%	74.3%	16.0%
	PMRS MGR	68.4%	63.2%	5.2%
	SMRS Control	89.6%	81.7%	7.9%
	SMRS Top	48.9%	44.1%	4.8%
	SMRS Bottom	76.9%	75.6%	1.3%
	SMRS MGR	72.2%	71.5%	0.7%
Naringenin	PMRS Control	24.6%	21.7%	2.9%
	PMRS Top	0.0%	0.0%	0.0%
	PMRS Bottom	2.4%	0.0%	2.4%
	PMRS MGR	2.4%	0.2%	2.2%
	SMRS Control	12.6%	12.0%	0.6%
	SMRS Top	0.0%	0.0%	0.0%
	SMRS Bottom	3.9%	3.5%	0.4%
	SMRS MGR	0.0%	7.2%	7.2%
Quercetin	PMRS Control	59.4%	48.2%	11.2%
	PMRS Top	29.2%	20.3%	9.0%
	PMRS Bottom	70.0%	70.1%	0.1%
	PMRS MGR	34.0%	25.0%	9.0%
	SMRS Control	90.8%	94.0%	3.2%
	SMRS Top	28.8%	22.0%	6.8%
	SMRS Bottom	81.9%	69.3%	12.6%
	SMRS MGR	73.4%	71.5%	1.9%
Melanin	PMRS Control	60.2%	57.5%	2.7%
	PMRS Top	9.5%	5.3%	4.2%
	PMRS Bottom	10.5%	11.1%	0.6%
	PMRS MGR	14.4%	10.6%	3.8%
	SMRS Control	55.0%	47.7%	7.3%
	SMRS Top	38.8%	35.5%	3.3%
	SMRS Bottom	22.8%	23.2%	0.4%
	SMRS MGR	34.2%	19.5%	14.7%
			**Average:**	**5.0%**

Table S3. Differences in signal coverage between anoxic and oxic sets according to the method (clustering or filtering).

		Anoxic	Oxic	Difference
Chlorophyllin			**Clustering**	
		Anoxic	Oxic	Difference
	PMRS Top	30.2%	23.6%	6.6%
	PMRS Bottom	90.6%	89.8%	0.8%
	SMRS Top	56.8%	41.0%	15.8%
	SMRS Bottom	81.8%	72.0%	9.8%
			**Filtering**	
		Anoxic	Oxic	Difference
	PMRS Top	39.0%	36.7%	2.3%
	PMRS Bottom	75.5%	73.0%	2.5%
	SMRS Top	51.8%	39.0%	12.8%
	SMRS Bottom	78.2%	73.0%	5.2%
Naringenin			**Clustering**	
		Anoxic	Oxic	Difference
	PMRS Top	0.0%	0.0%	0.0%
	PMRS Bottom	0.0%	4.8%	-4.8%
	SMRS Top	0.0%	0.0%	0.0%
	SMRS Bottom	0.2%	7.6%	-7.4%
			**Filtering**	
		Anoxic	Oxic	Difference
	PMRS Top	0.0%	0.0%	0.0%
	PMRS Bottom	0.0%	0.0%	0.0%
	SMRS Top	0.0%	0.0%	0.0%
	SMRS Bottom	0.0%	10.5%	-10.5%
Quercetin			**Clustering**	
		Anoxic	Oxic	Difference
	PMRS Top	35.6%	22.8%	12.8%
	PMRS Bottom	61.0%	79.0%	-18.0%
	SMRS Top	17.0%	40.6%	-23.6%
	SMRS Bottom	77.8%	86.0%	-8.2%
			**Filtering**	
		Anoxic	Oxic	Difference
	PMRS Top	17.8%	22.7%	-4.8%
	PMRS Bottom	66.2%	74.0%	-7.8%
	SMRS Top	23.3%	20.7%	2.7%
	SMRS Bottom	74.0%	93.0%	-19.0%
Melanin			**Clustering**	
		Anoxic	Oxic	Difference
	PMRS Top	12.2%	6.8%	5.4%
	PMRS Bottom	16.2%	4.8%	11.4%
	SMRS Top	60.4%	17.2%	43.2%
	SMRS Bottom	34.4%	11.2%	23.2%
			**Filtering**	
		Anoxic	Oxic	Difference
	PMRS Top	7.5%	3.6%	3.9%
	PMRS Bottom	10.1%	12.1%	-2.0%
	SMRS Top	46.5%	20.8%	25.7%
	SMRS Bottom	29.4%	14.3%	15.1%

REFERENCES AND NOTES

1. J. L. Eigenbrode, R. E. Summons, A. Steele, C. Freissinet, M. Millan, R. Navarro-González, B. Sutter, A. C. McAdam, H. B. Franz, D. P. Glavin, P. D. Archer, P. R. Mahaffy, P. G. Conrad, J. A. Hurowitz, J. P. Grotzinger, S. Gupta, D. W. Ming, D. Y. Sumner, C. Szopa, C. Malespin, A. Buch, P. Coll, Organic matter preserved in 3-billion-year-old mudstones at Gale crater, Mars. *Science* **360**, 1096–1101 (2018).

2. C. R. Webster, P. R. Mahaffy, S. K. Atreya, J. E. Moores, G. J. Flesch, C. Malespin, C. P. McKay, G. Martinez, C. L. Smith, J. Martin-Torres, J. Gomez-Elvira, M.-P. Zorzano, M. H. Wong, M. G. Trainer, A. Steele, D. Archer, B. Sutter, P. J. Coll, C. Freissinet, P.-Y. Meslin, R. V. Gough, C. H. House, A. Pavlov, J. L. Eigenbrode, D. P. Glavin, J. C. Pearson, D. Keymeulen, L. E. Christensen, S. P. Schwenzer, R. Navarro-Gonzalez, J. Pla-García, S. C. R. Rafkin, Á. Vicente-Retortillo, H. Kahanpää, D. Viudez-Moreiras, M. D. Smith, A.-M. Harri, M. Genzer, D. M. Hassler, M. Lemmon, J. Crisp, S. P. Sander, R. W. Zurek, A. R. Vasavada, Background levels of methane in Mars' atmosphere show strong seasonal variations. *Science* **360**, 1093–1096 (2018).

3. F. Rull, S. Maurice, I. Hutchinson, A. Moral, C. Perez, C. Diaz, M. Colombo, T. Belenguer, G. Lopez-Reyes, A. Sansano, O. Forni, Y. Parot, N. Striebig, S. Woodward, C. Howe, N. Tarcea, P. Rodriguez, L. Seoane, A. Santiago, J. A. Rodriguez-Prieto, J. Medina, P. Gallego, R. Canchal, P. Santamaría, G. Ramos, J. L. Vago; on behalf of the RLS Team, The Raman laser spectrometer for the ExoMars rover mission to Mars. *Astrobiology* **17**, 627–654 (2017).

4. L. Beegle, R. Bhartia, M. White, L. DeFlores, W. Abbey, Y.-H. Wu, B. Cameron, J. Moore, M. Fries, A. Burton, K. S. Edgett, M. A. Ravine, W. Hug, R. Reid, T. Nelson, S. Clegg, R. Wiens, S. Asher, P. Sobron, SHERLOC: Scanning habitable environments with Raman luminescence for organics chemicals, in *2015 IEEE Aerospace Conference* (IEEE, 7 to 14 March 2015), pp. 1–11.

5. R. C. Wiens, S. Maurice, K. Mccabe, P. Cais, R. B. Anderson, O. Beyssac, L. Bonal, S. Clegg, L. Deflores, G. Dromart, W. W. Fischer, O. Forni, O. Gasnault, J. P. Grotzinger, J. R. Johnson, J. Martinez-Frias, N. Mangold, S. Mclennan, F. Montmessin, F. Rull, S. K. Sharma, V. Sautter, E. Lewin, E. Cloutis, F. Poulet, S. Bernard, T. Mcconnochie, N. Lanza, H. Newsom, A. Ollila, R. Leveille, S. L. Mouélic, J. Lasue, N. Melikechi, P.-Y. Meslin, A. Misra, O. Grasset, S. M. Angel, T.

Fouchet, P. Beck, N. Bridges, B. Bousquet, C. Fabre, P. Pinet, K. Benzerara, G. Montagnac, The SuperCam remote sensing instrument suite for Mars 2020, in *47th Lunar and Planetary Science Conference* (2016), p. 1322.

6. A. Ellery, D. Wynn-Williams, Why Raman spectroscopy on Mars?—A case of the right tool for the right job. *Astrobiology* **3**, 565–579 (2003).

7. F. Foucher, M.-R. Ammar, F. Westall, Revealing the biotic origin of silicified Precambrian carbonaceous microstructures using Raman spectroscopic mapping, a potential method for the detection of microfossils on Mars. *J. Raman Spectrosc.* **46**, 873–879 (2015).

8. A. A. Pavlov, G. Vasilyev, V. M. Ostryakov, A. K. Pavlov, P. Mahaffy, Degradation of the organic molecules in the shallow subsurface of Mars due to irradiation by cosmic rays. *Geophys. Res. Lett.* **39**, L13202 (2012).

9. R. G. Keil, L. M. Mayer, Mineral Matrices and Organic Matter, in *Treatise on Geochemistry (Second Edition)*, H. D. Holland, K. K. Turekian, Eds. (Elsevier, 2014), pp. 337–359.

10. U. Böttger, J.-P. de Vera, J. Fritz, I. Weber, H.-W. Hübers, D. Schulze-Makuch, Optimizing the detection of carotene in cyanobacteria in a martian regolith analogue with a Raman spectrometer for the ExoMars mission. *Planet. Space Sci.* **60**, 356–362 (2012).

11. H. G. M. Edwards, I. B. Hutchinson, R. Ingley, J. Jehlička, Biomarkers and their Raman spectroscopic signatures: A spectral challenge for analytical astrobiology. *Philos. Trans. A Math. Phys. Eng. Sci.* **372**, 20140193 (2014).

12. M. Bertrand, A. Chabin, C. Colas, M. Cadène, D. Chaput, A. Brack, H. Cottin, F. Westall, The AMINO experiment: Exposure of amino acids in the EXPOSE-R experiment on the international space station and in laboratory. *Int. J. Astrobiology* **14**, 89–97 (2015).

13. F. Stalport, L. Rouquette, O. Poch, T. Dequaire, N. Chaouche-Mechidal, S. Payart, C. Szopa, P. Coll, D. Chaput, M. Jaber, F. Raulin, H. Cottin, The photochemistry on space station (PSS) experiment: Organic matter under mars-like surface UV radiation conditions in low earth orbit. *Astrobiology* **19**, 1037–1052 (2019).

14. H. Cottin, J. M. Kotler, D. Billi, C. Cockell, R. Demets, P. Ehrenfreund, A. Elsaesser, L. d'Hendecourt, J. J. W. A. van Loon, Z. Martins, S. Onofri, R. C. Quinn, E. Rabbow, P. Rettberg, A. J. Ricco, K. Slenzka, R. de la Torre, J.-P. de Vera, F. Westall, N. Carrasco, A. Fresneau, Y. Kawaguchi, Y. Kebukawa, D. Nguyen, O. Poch, K. Saiagh, F. Stalport, A. Yamagishi, H. Yano, B. A. Klamm, Space as a tool for astrobiology: Review and recommendations for experimentations in earth orbit and beyond. *Space Sci. Rev.* **209**, 83–181 (2017).

15. E. Rabbow, P. Rettberg, A. Parpart, C. Panitz, W. Schulte, F. Molter, E. Jaramillo, R. Demets, P. Weiß, R. Willnecker, EXPOSE-R2: The astrobiological ESA mission on board of the international space station. *Front. Microbiol.* **8**, 1533 (2017).

16. J.-P. de Vera, M. Alawi, T. Backhaus, M. Baqué, D. Billi, U. Böttger, T. Berger, M. Bohmeier, C. Cockell, R. Demets, R. de la Torre Noetzel, H. Edwards, A. Elsaesser, C. Fagliarone, A. Fiedler, B. Foing, F. Foucher, J. Fritz, F. Hanke, T. Herzog, G. Horneck, H.-W. Hübers, B. Huwe, J. Joshi, N. Kozyrovska, M. Kruchten, P. Lasch, N. Lee, S. Leuko, T. Leya, A. Lorek, J. Martínez-Frías, J. Meessen, S. Moritz, R. Moeller, K. Olsson-Francis, S. Onofri, S. Ott, C. Pacelli, O. Podolich, E. Rabbow, G. Reitz, P. Rettberg, O. Reva, L. Rothschild, L. G. Sancho, D. Schulze-Makuch, L. Selbmann, P. Serrano, U. Szewzyk, C. Verseux, J. Wadsworth, D. Wagner, F. Westall, D. Wolter, L. Zucconi, Limits of life and the habitability of Mars: The ESA space experiment BIOMEX on the ISS. *Astrobiology* **19**, 145–157 (2019).

17. J.-P. de Vera, U. Böttger, R. de la T. Noetzel, F. J. Sánchez, D. Grunow, N. Schmitz, C. Lange, H.-W. Hübers, D. Billi, M. Baqué, P. Rettberg, E. Rabbow, G. Reitz, T. Berger, R. Möller, M. Bohmeier, G. Horneck, F. Westall, J. Jänchen, J. Fritz, C. Meyer, S. Onofri, L. Selbmann, L. Zucconi, N. Kozyrovska, T. Leya, B. Foing, R. Demets, C. S. Cockell, C. Bryce, D. Wagner, P. Serrano, H. G. M. Edwards, J. Joshi, B. Huwe, P. Ehrenfreund, A. Elsaesser, S. Ott, J. Meessen, N. Feyh, U. Szewzyk, R. Jaumann, T. Spohn, Supporting Mars exploration: BIOMEX in low earth orbit and further astrobiological studies on the moon using Raman and PanCam technology. *Planet. Space Sci.* **74**, 103–110 (2012).

18. J. De Gelder, K. De Gussem, P. Vandenabeele, L. Moens, Reference database of Raman spectra of biological molecules. *J. Raman Spectrosc.* **38**, 1133–1147 (2007).

19. J. Jehlička, H. G. M. Edwards, A. Oren, Raman spectroscopy of microbial pigments. *Appl. Environ. Microbiol.* **80**, 3286–3295 (2014).

20. P. Hildebrandt, T. G. Spiro, Surface-enhanced resonance Raman spectroscopy of copper chlorophyllin on silver and gold colloids. *J. Phys. Chem.* **92**, 3355–3360 (1988).

21. O. Unsalan, Y. Erdogdu, M. T. Gulluoglu, FT-Raman and FT-IR spectral and quantum chemical studies on some flavonoid derivatives: Baicalein and Naringenin. *J. Raman Spectrosc.* **40**, 562–570 (2009).

22. T. Teslova, C. Corredor, R. Livingstone, T. Spataru, R. L. Birke, J. R. Lombardi, M. V. Cañamares, M. Leona, Raman and surface-enhanced Raman spectra of flavone and several hydroxy derivatives. *J. Raman Spectrosc.* **38**, 802–818 (2007).

23. Z. Huang, H. Lui, X. K. Chen, A. Alajlan, D. I. McLean, H. Zeng, Raman spectroscopy of in vivo cutaneous melanin. *J. Biomed. Opt.* **9**, 1198–1205 (2004).

24. R. C. Wiens, S. Maurice, F. R. Perez, The SuperCam remote sensing instrument suite for the Mars 2020 rover mission: A preview. *Spectroscopy* **32**, (2017).

25. C. S. Cockell, D. C. Catling, W. L. Davis, K. Snook, R. L. Kepner, P. Lee, C. P. McKay, The ultraviolet environment of Mars: Biological implications past, present, and future. *Icarus* **146**, 343–359 (2000).

26. D. M. Hassler, C. Zeitlin, R. F. Wimmer-Schweingruber, B. Ehresmann, S. Rafkin, J. L. Eigenbrode, D. E. Brinza, G. Weigle, S. Böttcher, E. Böhm, S. Burmeister, J. Guo, J. Köhler, C. Martin, G. Reitz, F. A. Cucinotta, M.-H. Kim, D. Grinspoon, M. A. Bullock, A. Posner, J. Gómez-Elvira, A. Vasavada, J. P. Grotzinger, M. S. Team, Mars' surface radiation environment measured with the mars science laboratory's curiosity rover. *Science* **343**, 1244797 (2014).

27. S. Leuko, M. Bohmeier, F. Hanke, U. Böttger, E. Rabbow, A. Parpart, P. Rettberg, J.-P. P. de Vera, On the stability of deinoxanthin exposed to mars conditions during a long-term space mission and implications for biomarker detection on other planets. *Front. Microbiol.* **8**, 1680 (2017).

28. C. C. Bryce, G. Horneck, E. Rabbow, H. G. M. Edwards, C. S. Cockell, Impact shocked rocks as protective habitats on an anoxic early Earth. *Int. J. Astrobiology* **14**, 115–122 (2015).

29. M. Baqué, F. Hanke, U. Böttger, T. Leya, R. Moeller, J.-P. de Vera, Protection of cyanobacterial carotenoids' Raman signatures by Martian mineral analogues after high-dose gamma irradiation. *J. Raman Spectrosc.* **49**, 1617–1627 (2018).

30. I. Orlovska, O. Podolich, O. Kukharenko, I. Zaets, O. Reva, L. Khirunenko, D. Zmejkoski, S. Rogalsky, D. Barh, S. Tiwari, R. Kumavath, A. Góes-Neto, V. Azevedo, B. Brenig, P. Ghosh, J.-P. de Vera, N. Kozyrovska, Bacterial cellulose retains robustness but its synthesis declines after exposure to a mars-like environment simulated outside the international space station. *Astrobiology* **21**, 706–717 (2021).

31. C. Pacelli, L. Selbmann, L. Zucconi, C. Coleine, J.-P. de Vera, E. Rabbow, U. Böttger, E. Dadachova, S. Onofri, Responses of the black fungus cryomyces antarcticus to simulated mars and space conditions on rock analogs. *Astrobiology* **19**, 209–220 (2019).

32. R. E. Summons, J. P. Amend, D. Bish, R. Buick, G. D. Cody, D. J. Des Marais, G. Dromart, J. L. Eigenbrode, A. H. Knoll, D. Y. Sumner, Preservation of martian organic and environmental records: Final report of the mars biosignature working group. *Astrobiology* **11**, 157–181 (2011).

33. F. Sauro, M. Cappelletti, D. Ghezzi, A. Columbu, P.-Y. Hong, H. M. Zowawi, C. Carbone, L. Piccini, F. Vergara, D. Zannoni, J. D. Waele, Microbial diversity and biosignatures of amorphous silica deposits in orthoquartzite caves. *Sci. Rep.* **8**, 17569 (2018).

34. C. Quantin-Nataf, J. Carter, L. Mandon, P. Thollot, M. Balme, M. Volat, L. Pan, D. Loizeau, C. Millot, S. Breton, E. Dehouck, P. Fawdon, S. Gupta, J. Davis, P. M. Grindrod, A. Pacifici, B. Bultel, P. Allemand, A. Ody, L. Lozach, J. Broyer, Oxia planum: The landing site for the exomars "Rosalind Franklin" rover mission: Geological context and prelanding interpretation. *Astrobiology* **21**, 345–366 (2021).

35. J. D. Tarnas, J. F. Mustard, H. Lin, T. A. Goudge, E. S. Amador, M. S. Bramble, C. H. Kremer, X. Zhang, Y. Itoh, M. Parente, Orbital identification of hydrated silica in Jezero crater, mars. *Geophys. Res. Lett.* **46**, 12771–12782 (2019).

36. F. Hanke, B. J. A. Mooij, F. Ariese, U. Böttger, The evaluation of time-resolved Raman spectroscopy for the suppression of background fluorescence from space-relevant samples. *J. Raman Spectrosc.* **50**, 969–982 (2019).

37. L. Demaret, I. B. Hutchinson, G. Eppe, C. Malherbe, Quantitative analysis of binary and ternary organo-mineral solid dispersions by Raman spectroscopy for robotic planetary exploration missions on Mars. *Analyst* **146**, 7306–7319 (2021).

38. J. L. Vago, F. Westall; Pasteur Instrument Teams, Landing Site Selection Working Group, and other contributors, A. J. Coates, R. Jaumann, O. Korablev, V. Ciarletti, I. Mitrofanov, J.-L. Josset, M. C. De Sanctis, J.-P. Bibring, F. Rull, F. Goesmann, H. Steininger, W. Goetz, W. Brinckerhoff, C. Szopa, F. Raulin, F. Westall, H. G. M. Edwards, L. G. Whyte, A. G. Fairén, J.-P. Bibring, J. Bridges, E. Hauber, G. G. Ori, S. Werner, D. Loizeau, R. O. Kuzmin, R. M. E. Williams, J. Flahaut, F. Forget, J. L. Vago, D. Rodionov, O. Korablev, H. Svedhem, E. Sefton-Nash, G. Kminek, L. Lorenzoni, L. Joudrier, V. Mikhailov, A. Zashchirinskiy, S. Alexashkin, F. Calantropio, A. Merlo, P. Poulakis, O. Witasse, O. Bayle, S. Bayón, U. Meierhenrich, J. Carter, J. M. García-Ruiz, P. Baglioni, A. Haldemann, A. J. Ball, A. Debus, R. Lindner, F. Haessig, D. Monteiro, R. Trautner, C. Voland, P. Rebeyre, D. Goulty, F. Didot, S. Durrant, E. Zekri, D. Koschny, A. Toni, G. Visentin, M. Zwick, M. van Winnendael, M. Azkarate, C. Carreau; and the ExoMars Project Team, Habitability on early mars and the search for biosignatures with the ExoMars rover. *Astrobiology* **17**, 471–510 (2017).

39. J. Wang, J. Tavakoli, Y. Tang, Bacterial cellulose production, properties and applications with different culture methods–A review. *Carbohydr. Polym.* **219**, 63–76 (2019).

40. K. Goiris, K. Muylaert, S. Voorspoels, B. Noten, D. D. Paepe, G. J. E. Baart, L. D. Cooman, Detection of flavonoids in microalgae from different evolutionary lineages. *J. Phycol.* **50**, 483–492 (2014).

41. S. Liu, J. Ju, G. Xia, Identification of the flavonoid 3′-hydroxylase and flavonoid 3′,5′-hydroxylase genes from Antarctic moss and their regulation during abiotic stress. *Gene* **543**, 145–152 (2014).

42. S. Onofri, J.-P. de Vera, L. Zucconi, L. Selbmann, G. Scalzi, K. J. Venkateswaran, E. Rabbow, R. de la Torre, G. Horneck, Survival of antarctic cryptoendolithic fungi in simulated martian conditions on board the international space station. *Astrobiology* **15**, 1052–1059 (2015).

43. J. Meeßen, F. J. Sánchez, A. Brandt, E.-M. Balzer, R. de la Torre, L. G. Sancho, J.-P. de Vera, S. Ott, Extremotolerance and resistance of lichens: Comparative studies on five species used in astrobiological research I. morphological and anatomical characteristics. *Orig. Life Evol. Biosph.* **43**, 283–303 (2013).

44. S. Nie, L. A. Lipscomb, N.-T. Yu, Surface-enhanced hyper-Raman spectroscopy. *Appl. Spectrosc. Rev.* **26**, 203–276 (1991).

45. N. Gueneli, A. M. McKenna, N. Ohkouchi, C. J. Boreham, J. Beghin, E. J. Javaux, J. J. Brocks, 1.1-billion-year-old porphyrins establish a marine ecosystem dominated by bacterial primary producers. *Proc. Natl. Acad. Sci. U.S.A.* **115**, E6978–E6986 (2018).

46. J. P. Grotzinger, D. Y. Sumner, L. C. Kah, K. Stack, S. Gupta, L. Edgar, D. Rubin, K. Lewis, J. Schieber, N. Mangold, R. Milliken, P. G. Conrad, D. DesMarais, J. Farmer, K. Siebach, F. Calef, J. Hurowitz, S. M. McLennan, D. Ming, D. Vaniman, J. Crisp, A. Vasavada, K. S. Edgett, M. Malin, D. Blake, R. Gellert, P. Mahaffy, R. C. Wiens, S. Maurice, J. A. Grant, S. Wilson, R. C. Anderson, L. Beegle, R. Arvidson, B. Hallet, R. S. Sletten, M. Rice, J. Bell, J. Griffes, B. Ehlmann, R. B. Anderson, T. F. Bristow, W. E. Dietrich, G. Dromart, J. Eigenbrode, A. Fraeman, C. Hardgrove, K. Herkenhoff, L. Jandura, G. Kocurek, S. Lee, L. A. Leshin, R. Leveille, D. Limonadi, J. Maki, S. McCloskey, M. Meyer, M. Minitti, H. Newsom, D. Oehler, A. Okon, M. Palucis, T. Parker, S. Rowland, M. Schmidt, S. Squyres, A. Steele, E. Stolper, R. Summons, A. Treiman, R. Williams, A. Yingst; MSL Science Team, A habitable fluvio-lacustrine environment at yellowknife bay, gale crater, Mars. *Science* **343**, 1242777 (2014).

47. C. S. Cockell, Trajectories of martian habitability. *Astrobiology* **14**, 182–203 (2014).

48. C. P. McKay, An origin of life on Mars. *Cold Spring Harb. Perspect. Biol.* **2**, a003509 (2010).

49. F. Westall, D. Loizeau, F. Foucher, N. Bost, M. Betrand, J. Vago, G. Kminek, Habitability on Mars from a microbial point of view. *Astrobiology* **13**, 887–897 (2013).

50. B. Haezeleer, U. Böttger, J.-P. de Vera, F. Hanke, S. Fox, H. Strasdeit, Artifact formation during Raman measurements and its relevance to the search for chemical biosignatures on Mars. *Planet. Space Sci.* **179**, 104714 (2019).

51. A. Culka, J. Jehlička, C. Ascaso, O. Artieda, C. M. Casero, J. Wierzchos, Raman microspectrometric study of pigments in melanized fungi from the hyperarid Atacama desert gypsum crust. *J. Raman Spectrosc.* **48**, 1487–1493 (2017).

52. P. Ehrenfreund, S. B. Charnley, Organic molecules in the interstellar medium, comets, and meteorites: A voyage from dark clouds to the early earth. *Annu. Rev. Astron. Astrophys.* **38**, 427–483 (2000).

53. K. A. Kvenvolden, J. G. Lawless, K. Pering, E. Peterson, J. Flores, C. Ponnamperuma, I. R. Kaplan, C. Moore, Evidence for extraterrestrial amino-acids and hydrocarbons in the murchison meteorite. *Nature* **228**, 923–926 (1970).

54. J. Oró, A. P. Kimball, Synthesis of purines under possible primitive earth conditions. I. Adenine from hydrogen cyanide. *Arch. Biochem. Biophys.* **94**, 217–227 (1961).

55. D. S. McKay, E. K. Gibson, K. L. Thomas-Keprta, H. Vali, C. S. Romanek, S. J. Clemett, X. D. F. Chillier, C. R. Maechling, R. N. Zare, Search for past life on Mars: Possible relic biogenic activity in martian meteorite ALH84001. *Science* **273**, 924–930 (1996).

56. G. D. Chumanov, R. G. Efremov, I. R. Nabiev, Surface-enhanced Raman spectroscopy of biomolecules. Part I.—Water-soluble proteins, dipeptides and amino acids. *J. Raman Spectrosc.* **21**, 43–48 (1990).

57. A. S. Burton, J. C. Stern, J. E. Elsila, D. P. Glavin, J. P. Dworkin, Understanding prebiotic chemistry through the analysis of extraterrestrial amino acids and nucleobases in meteorites. *Chem. Soc. Rev.* **41**, 5459–5472 (2012).

58. I. L. ten Kate, J. R. Garry, Z. Peeters, R. Quinn, B. Foing, P. Ehrenfreund, Amino acid photostability on the Martian surface. *Meteorit. Planet. Sci.* **40**, 1185–1193 (2005).

59. P. Vandenabeele, J. Jehlička, P. Vítek, H. G. M. Edwards, On the definition of Raman spectroscopic detection limits for the analysis of biomarkers in solid matrices. *Planet. Space Sci.* **62**, 48–54 (2012).

60. Y. D. Winters, T. K. Lowenstein, M. N. Timofeeff, Identification of carotenoids in ancient salt from Death Valley, Saline Valley, and Searles Lake, California, using laser Raman spectroscopy. *Astrobiology* **13**, 1065–1080 (2013).

61. C. Lee, J. J. Brocks, Identification of carotane breakdown products in the 1.64 billion year old Barney Creek Formation, McArthur Basin, northern Australia. *Org. Geochem.* **42**, 425–430 (2011).

62. F. Westall, F. Foucher, N. Bost, M. Bertrand, D. Loizeau, J. L. Vago, G. Kminek, F. Gaboyer, K. A. Campbell, J.-G. Bréhéret, P. Gautret, C. S. Cockell, Biosignatures on Mars: What, where, and how? Implications for the search for martian life. *Astrobiology* **15**, 998–1029 (2015).

63. J. Alcaíno, M. Baeza, V. Cifuentes, Carotenoid Distribution in Nature, in *Carotenoids in Nature*, C. Stange, Ed. (Springer International Publishing, 2016), vol. 79, pp. 3–33.

64. W. Stahl, H. Sies, Antioxidant activity of carotenoids. *Mol. Aspects Med.* **24**, 345–351 (2003).

65. C. S. Cockell, The Ultraviolet Radiation Environment of Earth and Mars: Past and Present, in *Astrobiology: The Quest for the Conditions of Life*, G. Horneck, C. Baumstark-Khan, Eds. (Springer, 2002), pp. 219–232.

66. G. Ourisson, Y. Nakatani, The terpenoid theory of the origin of cellular life: The evolution of terpenoids to cholesterol. *Chem. Biol.* **1**, 11–23 (1994).

67. C. Pacelli, A. Cassaro, A. Maturilli, A. M. Timperio, F. Gevi, B. Cavalazzi, M. Stefan, D. Ghica, S. Onofri, Multidisciplinary characterization of melanin pigments from the black fungus *Cryomyces antarcticus*. *Appl. Microbiol. Biotechnol.* **104**, 6385–6395 (2020).

68. J. Meeßen, P. Wuthenow, P. Schille, E. Rabbow, J.-P. P. de Vera, S. Ott, Resistance of the lichen *Buellia frigida* to simulated space conditions during the preflight tests for BIOMEX—Viability assay and morphological stability. *Astrobiology* **15**, 601–615 (2015).

69. M. d'Ischia, P. Manini, Z. Martins, L. Remusat, C. M. O'D. Alexander, C. Puzzarini, V. Barone, R. Saladino, Insoluble organic matter in chondrites: Archetypal melanin-like PAH-based multifunctionality at the origin of life? *Phys. Life Rev.* **37**, 65–93 (2021).

70. A. Brandt, E. Posthoff, J.-P. de Vera, S. Onofri, S. Ott, Characterisation of growth and ultrastructural effects of the *Xanthoria elegans* photobiont after 1.5 years of space exposure on the international space station. *Orig. Life Evol. Biosph.* **46**, 1–11 (2015).

71. Z. Suo, R. Avci, M. H. Schweitzer, M. Deliorman, Porphyrin as an ideal biomarker in the search for extraterrestrial life. *Astrobiology* **7**, 605–615 (2007).

72. S. Fox, H. Strasdeit, A possible prebiotic origin on volcanic islands of oligopyrrole-type photopigments and electron transfer cofactors. *Astrobiology* **13**, 578–595 (2013).

73. A. W. Larkum, The evolution of chlorophylls and photosynthesis, in *Chlorophylls and bacteriochlorophylls* (Springer, 2006), pp. 261–282.

74. D. Klemm, B. Heublein, H.-P. Fink, A. Bohn, Cellulose: Fascinating biopolymer and sustainable raw material. *Angew. Chem. Int. Ed.* **44**, 3358–3393 (2005).

75. K. Ogawa, Y. Maki, Cellulose as extracellular polysaccharide of hot spring sulfur-turf bacterial Mat. *Biochemistry* **67**, 2652–2654 (2003).

76. D. R. Nobles, R. M. Brown, The pivotal role of cyanobacteria in the evolution of cellulose synthases and cellulose synthase-like proteins. *Cellul.* **11**, 437–448 (2004).

77. N. Kato, T. Sato, C. Kato, M. Yajima, J. Sugiyama, T. Kanda, M. Mizuno, K. Nozaki, S. Yamanaka, Y. Amano, Viability and cellulose synthesizing ability of *Gluconacetobacter xylinus* cells under high-hydrostatic pressure. *Extremophiles* **11**, 693–698 (2007).

78. J. D. Griffith, S. Willcox, D. W. Powers, R. Nelson, B. K. Baxter, Discovery of abundant cellulose microfibers encased in 250 Ma Permian halite: A macromolecular target in the search for life on other planets. *Astrobiology* **8**, 215–228 (2008).

79. D. E. Briggs, R. E. Summons, Ancient biomolecules: Their origins, fossilization, and role in revealing the history of life. *Bioessays* **36**, 482–490 (2014).

80. H. Ehrlich, Chitin and collagen as universal and alternative templates in biomineralization. *Int. Geol. Rev.* **52**, 661–699 (2010).
